# Supplementary figures and images for: Filovirus receptor NPC1 contributes to species-specific patterns of ebolavirus susceptibility in bats
Source: eLife. 2015 Dec 23;4:e11785. doi: 10.7554/eLife.11785 (PMC4709267; doi:10.7554/eLife.11785)

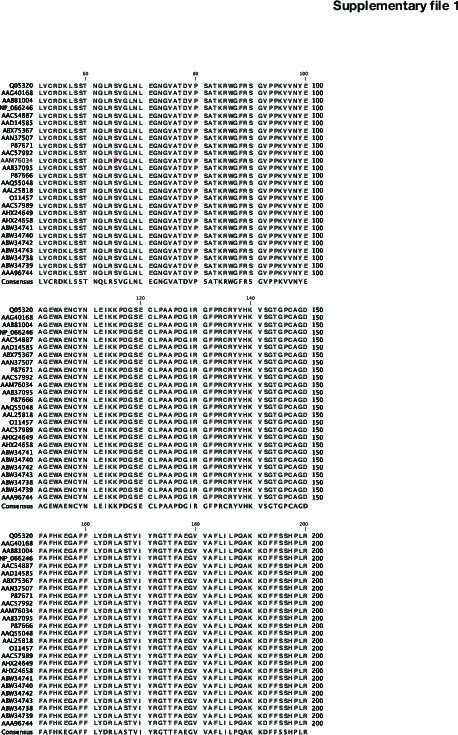

Supplement: Supplementary file 1. — Alignment of GP amino acid sequences corresponding to the NPC1-binding site (residues 53-200) derived from diverse EBOV isolates (listed GenBank accession numbers) is shown. Amino acid changes are highlighted in pink. DOI: http://dx.doi.org/10.7554/eLife.11785.017 [file elife-11785-supp1.jpg]

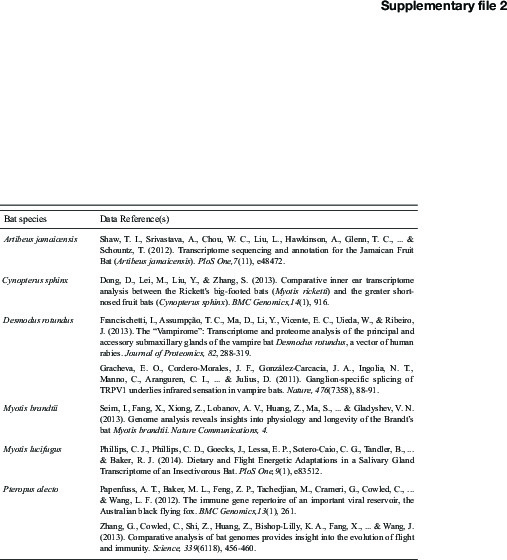

Supplement: Supplementary file 2. — DOI: http://dx.doi.org/10.7554/eLife.11785.018 [file elife-11785-supp2.jpg]

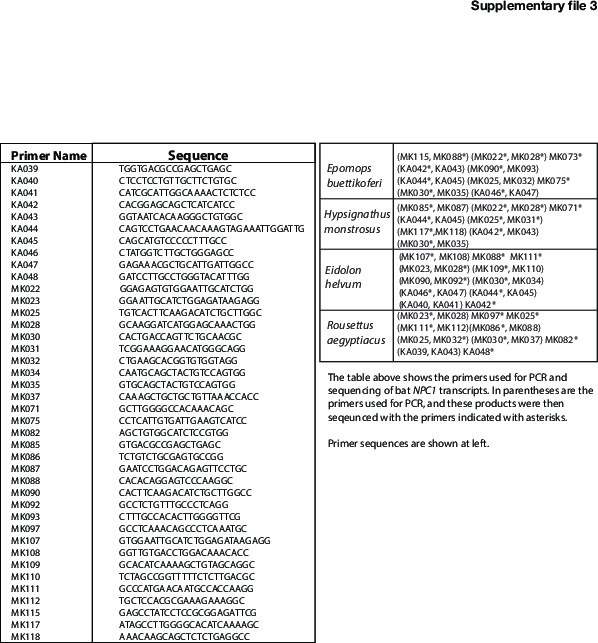

Supplement: Supplementary file 3. — DOI: http://dx.doi.org/10.7554/eLife.11785.019 [file elife-11785-supp3.jpg]
