# Supplementary material for: Filovirus receptor NPC1 contributes to species-specific patterns of ebolavirus susceptibility in bats
Source: eLife. 2015 Dec 23;4:e11785. doi: 10.7554/eLife.11785 (PMC4709267; doi:10.7554/eLife.11785)
Supplement: Supplementary file 4. — DOI: http://dx.doi.org/10.7554/eLife.11785.020 [file elife-11785-supp4.zip › Supplementary file 4.rtf]

Epomops_beuttikoferi         ATGAGTGCGCGCGGCCGGGTCCTGGGCCTCCTCCTGTTCCTGCTGTGCCTEidolon_helvum               ATGAATGCGCGCGGCCCGGCCCTGGGCCTCCTCCTGTTGCTGCTGTGCCTHypsignathus_monstrosus      ATGAGTGCGCGCGGCCGGGTCCTGGGCCTCCTCCTGTTCCTGCTGTGCCTRousettus_aegyptiacus        ATGAGTGCACGCGGCCGGGTCCTGGGCCTCCTCCTGTTGCTGCTGTGCCCMyotis_lucifugus             ATGAGTGCGCGCGGCCTGGCCCTGGGC---CTCCTGCTGCTGCTGTGCCCMyotis_brandtii              ATGAGTGCGCGCGGCCTGACCCTGGGC---CTCCTGCTGCTGCTGTGCCCPteropus_alecto              ATGGGTGCGCGCGGCCCGGCCCTGGGCCTCCTCCTGTTGCTGCTGTGCCCArtibeus_jamaicensis         ATGAGTGCGCGCGGCCTGACACTGGGCCTACTCTTGTTGCTGCT---CCCCynopterus_sphinx            ------------------------------------------CTGTGCCCDesmodus_rotundus            ATGAGTGCGCGCGGCCTGGCACTGGGCCTACTCTTGTTGCTGCTGTGCCC                                                                       **   ** Epomops_beuttikoferi         GGCGCAAGTGTTTTCACAGTCCTGCGTTTGGTATGGAGAGTGTGGAATTGEidolon_helvum               GGCGCAGGTGTTTTCACAGTCCTGTGTTTGGTATGGAGAGTGTGGAATTGHypsignathus_monstrosus      GGCGCAAGTGTTTTCACAGTCCTGCGTTTGGTATGGAGAGTGTGGAATTGRousettus_aegyptiacus        GGCGCAAGTGTTTTCACAGTCCTGTGTTTGGTATGGAGAGTGTGGAATTGMyotis_lucifugus             GGCGCAGGTGTTTCCGCAGTCCTGTGTCTGGTATGGAGAGTGTGGGATTGMyotis_brandtii              GGCGCAGGTGTTTCCGCAGTCCTGCGTCTGGTATGGAGAGTGTGGGATTGPteropus_alecto              GGCGCAGGTGTTTTCACAGTCCTGTGTTTGGTATGGAGAGTGTGGAATTGArtibeus_jamaicensis         GGCACAGGCATTTTCACAGTCCTGCGTTTGGTATGGAGAATGTGGAATTGCynopterus_sphinx            CGCGCAGGTATTTTCACAGTCCTGTGTTTGGTATGGAGAGTGTGGAATTGDesmodus_rotundus            GGCACAGGCATTTTCACAGTCCTGTGTTTGGTATGGAGAATGCGGAATTG                              ** ** *  *** * ******** ** *********** ** ** ****Epomops_beuttikoferi         CATCTGGAGATAAGAGGTACAATTGCAAATATTCTGGACCACCAAAACCTEidolon_helvum               CATCTGGAGATAAGAGGTACAATTGCAAATATTCTGGACTACCAAAATCTHypsignathus_monstrosus      CATCTGGAGATAAGAGGTACAATTGCAAATATTCTGGACCACCAAAACCTRousettus_aegyptiacus        CATCTGGAGATAAGAGGTACAATTGCAAATATTCTGGAAAACCAAAACTTMyotis_lucifugus             CTTCTGGAGACAAGAGGTACAACTGCGAGTATTCCGGGCCCCCGAAGGCAMyotis_brandtii              CTTCTGGAGACAAGAGGTACAACTGCGAGTATTCCGGGCCCCCGCAGGCAPteropus_alecto              CATCTGGAGATAAGAGGTACAATTGCAAATATTCTGGACCACCATTGCCTArtibeus_jamaicensis         CATCTGGAGATAAGAGGTACAACTGCAAATATTCTGGGCCACCAAAAGCACynopterus_sphinx            CATCTGGAGATAAGAGGTACAATTGCAAATATTCTGGACTACCAAAACATDesmodus_rotundus            CGTCTGGAGATAAGAGGTACAATTGCAAATATTCTGGGCCACCAAAAACA                             * ******** *********** *** * ***** **    **       Epomops_beuttikoferi         TTGCCAAAAGATGGTTATGACTTAGTGCAGGAACTCTGTCCAGGATTCTTEidolon_helvum               TTGCCAAAAGATGGTTATGACTTAGTACAGGAACTCTGTCCAGGATTCTTHypsignathus_monstrosus      TTGCCAAAAGATGGTTATGACTTAGTGCAGGAACTCTGTCCAGGATTCTTRousettus_aegyptiacus        TTGCCAGAAGATGGTTATGACTTAGTGCAGGAACTCTGTCCAGGATTCTTMyotis_lucifugus             CTGCCGAAGGACGGGTATGACTTAGTGCAGGAACTCTGTCCAGGGCTCTTMyotis_brandtii              CTGCCGAAGGACGGGTATGACTTAGTGCAGGAACTCTGTCCGGGGTTCTTPteropus_alecto              TTGCCAAAAGATGGTTATGACTTAGTGCAGGAACTCTGTCCAGGATTCTTArtibeus_jamaicensis         TTGCCAGAGGATGGGTATGACTTAGTGCAGGAACTCTGTCCAGGCTTCTTCynopterus_sphinx            TTGCCAAAAGATGGTTATGACTTAGTGCAGGAACTCTGTCCAGGATTCTTDesmodus_rotundus            TTGCCAAAGGATGGGTATGACTTGGTGCAGGAACTCTGTCCAGGCTTCTT                              ****  * ** ** ******** ** ************** **  ****Epomops_beuttikoferi         CTTTGGCAATGTCAGTCTTTGTTGTGATGTTCAGCAGCTTCAGACACTGAEidolon_helvum               CTTTGACAATGTCAGTCTTTGTTGTGATGTTCAGCAGCTTCGGACACTGAHypsignathus_monstrosus      CTTTGGCAATGTCAGTCTTTGTTGTGATGTTCAGCAGCTTCAGACACTGARousettus_aegyptiacus        CTTTGGCAATGTCAGTCTTTGTTGTGATGTTCAGCAGCTTCGGACATTGAMyotis_lucifugus             CTTCGGCAATGTCAGCCTCTGCTGTGATGTGCAGCAGCTCCGGACCCTGAMyotis_brandtii              CTTCGGCAACGTCAGCCTCTGCTGTGATGTGCAGCAGCTCCGGACCCTGAPteropus_alecto              TTTTGGCAATATCAGTCTTTGTTGTGATGTTCAGCAGCTTCAGACACTGAArtibeus_jamaicensis         CTTTGACAATGTCAGTCTTTGTTGTGATGTTCAGCAGCTTCAGACACTGACynopterus_sphinx            CTTTGACAATGTCAGCCTTTGTTGTGATGTTCAGCAGCTTCGGACACTGADesmodus_rotundus            CTTTGACAACGTCAGTCTTTGCTGCGACGTCCAGCAGCTGCGGACCCTGA                              ** * ***  **** ** ** ** ** ** ******** * ***  ***Epomops_beuttikoferi         AAGACAGCCTGCAGCTGCCTCTACAGTTTCTGTCCAGATGTCCTTCCTGTEidolon_helvum               AAGACAGCCTGCAGCTGCCTCTACAGTTTCTGTCCAGATGTCCTTCCTGTHypsignathus_monstrosus      AAGACAGCCTGCAGCTGCCTCTACAGTTTCTGTCCAGATGTCCTTCCTGTRousettus_aegyptiacus        AAGACAGCCTGCAGCTGCCTCTACAGTTTCTGTCCAGATGTCCTTCCTGTMyotis_lucifugus             AAGACAACCTGCAGCTGCCTCTCCAGTTTCTGTCCAGATGTCCATCCTGTMyotis_brandtii              AAGACAACCTGCAGCTGCCTCTCCAGTTTCTGTCCAGATGTCCATCCTGTPteropus_alecto              AAGACAACCTGCAGCTGCCTCTCCAGTTTCTGTCCAGATGTCCTTCCTGTArtibeus_jamaicensis         AAGACAGCCTGCAGCTGCCTCTACAGTTTCTGTCCAGATGTCCATCGTGCCynopterus_sphinx            AAGACAACCTGCAGCTGCCTCTACAGTTTCTGTCCAGATGTCCTTCCTGTDesmodus_rotundus            AAGACAACCTGCAGCTGCCTCTGCAGTTTCTGTCCAGATGTCCGTCCTGT                             ****** *************** ******************** ** ** Epomops_beuttikoferi         TTTTATAACCTACTGAACATGTTTTGTGAGCTGACATGTAGCCCTCGACAEidolon_helvum               TTTTACAACCTACTGAACATGTTTTGTGAGCTGACATGTAGCCCTCGACAHypsignathus_monstrosus      TTTTATAACCTACTGAACATGTTTTGTGAGCTGACATGTAGCCCTCGACARousettus_aegyptiacus        TTTTATAACCTACTGAACATGTTTTGTGAGCTGACATGTAGCCCTAGACAMyotis_lucifugus             TTTTATAACCTGATGAACTTGTTTTGTGAGCTGACGTGTAGCCCTCGACAMyotis_brandtii              TTTTATAACCTGATGAACTTGTTTTGTGAGCTGACATGTAGCCCTCGACAPteropus_alecto              TTTTATAACCTACTGAACATGTTTTGTGAGCTGACATGTAGCCCTCGACAArtibeus_jamaicensis         TTTTATAACCTAATGAACCTGTTTTGTGAGCTGACATGTAGCCCTCGGCACynopterus_sphinx            TTTTATAACCTACTGAACATGTTTTGTGAGCTGACATGTAGCCCTCGACADesmodus_rotundus            TTTTATAACCTAATGAACCTGTTCTGTGAGCTGACGTGTAGCCCTCGGCA                             ***** *****  ***** **** *********** ********* * **Epomops_beuttikoferi         AAGTGAGTTTCTGAACATTACAGCAACTGAAGATTATGTTGATCCTGTTAEidolon_helvum               AAGTGAGTTTCTGAACGTTACAGCAACTGAAGATTATTTTGATCCTGTTAHypsignathus_monstrosus      AAGTGAGTTTCTGAACATTACAGCAACTGAAGATTATGTTGATCCTGTTARousettus_aegyptiacus        AAGTGAGTTTCTGAACATTACAGCAACTGAAGATTATGTCGATCCTGTTAMyotis_lucifugus             AAGCCAGTTTCTGAACGTTACAGAAACCGAAGAGTATGTGGACCCTGTTAMyotis_brandtii              AAGCCAGTTTCTGAACGTTACAGAAACCGAAGAGTATGTGGACCCTGTTAPteropus_alecto              AAGTGAGTTTCTGAACGTTACAGCAACTGAAGATTATTTTGATCCTGTTAArtibeus_jamaicensis         AAGTCAGTTTCTAAACGTTACAGAAACTGAAGATTATGTTGATCCTGTTACynopterus_sphinx            AAGTGAGTTTCTGAACGTTACAGCAACTGAAGATTATGTTGATCCTGTTADesmodus_rotundus            AAGTCAGTTTCTAAACGTTACAGAAACTGGAGATTATGTTGATCCTGTTA                             ***  ******* *** ****** *** * *** *** * ** *******Epomops_beuttikoferi         CAAATCAGACAAAAATTAATGTAAATGAATTGCAATACTATCTTGGAGAGEidolon_helvum               TAAATCACACGAAAACTAATGTAAAAGAATTGCAGTACTATGTTGGAGAGHypsignathus_monstrosus      CAAATCAGACAAAAATTAATGTAAATGAATTGCAATACTATCTTGGAGAGRousettus_aegyptiacus        TAAATCAGACAAAAACTAATGTAAAAGAATTGCAGTACTATATTGGAGAGMyotis_lucifugus             CCCACGAGAATAAAACAAACGTGAAGGAGTTACAGTACTATGTTGGAGAGMyotis_brandtii              CCCACGAGAATAAAACAAACGTGAAGGAGTTGCAGTACTATGTTGGAGAGPteropus_alecto              TACATGAGACGAAAACTAATGTAAAAGAATTGCAGTACTATGTTGGAGAGArtibeus_jamaicensis         CACACCAGATGAAAACAAATGTAAAAGAATTACAGTACTATGTCGGAGAGCynopterus_sphinx            CAAATCTGACGAAAACTAATGTAAAAGAATTACAGTACTATATTGGAGAGDesmodus_rotundus            CAAACCAGACGAAAACAAACGTAGAAGAATTACAGTACTATGTTGGAGAG                                *    *  ****  ** **  * ** ** ** ****** * ******Epomops_beuttikoferi         AGTTTTGCCAATGCGATGTACAATGCCTGCAGGGACGTGGAGGCCCCCTCEidolon_helvum               AGTTTTGCCAATGCAATGTACAATGCCTGCAGGGACGTGGAGGCCCCCTCHypsignathus_monstrosus      AGTTTTGCCAATGCGATGTACAATGCCTGCAGGGACGTGGAGGCCCCCTCRousettus_aegyptiacus        AGTTTTGCCAATGCGATGTACAATGCCTGCAGGGACGTGGAGGCCCCCTCMyotis_lucifugus             AGTTTTGCCAATGCCATGTACAACGCCTGCAGGGACGTGGAGGCCCCCTCMyotis_brandtii              AGTTTTGCCAATGCCATGTACAACGCCTGCAGGGACGTGGAGGCCCCCTCPteropus_alecto              AGTTTTGCCAATGCGATGTACAATGCCTGCAGGGACGTGGAGGCCCCCTCArtibeus_jamaicensis         AGTTTTGCCAATGCGATGTACAACGCCTGCAGGGACGTGGAGGCCCCCTCCynopterus_sphinx            AGTTTTGCCAATGCGATGTACAATGCCTGCAGGGACGTGGAGGCCCCCTCDesmodus_rotundus            AGTTTTGCCAATGCCATGTACAACGCCTGCAGGGACGTGGAGGCCCCCTC                             ************** ******** **************************Epomops_beuttikoferi         AAGTAATGACAAAGCCTTGGGACTCCTGTGTGGGAAGGACGCCGAAGCCTEidolon_helvum               AAGTAATGACAAAGCCTTGGGACTCCTGTGTGGGAAGGACGCTGAAGCCTHypsignathus_monstrosus      AAGTAATGACAAAGCCTTGGGACTCCTGTGTGGGAAGGACGCCGAAGCCTRousettus_aegyptiacus        AAGTAATGACAAAGCCTTGGGACTCCTGTGTGGGAAGGACGCCAAAGCCTMyotis_lucifugus             GAGCAACGACAAGGCCCTGGGGCTCCTGTGTGGGAAGGAGGCCGCAGCCTMyotis_brandtii              GAGCAACGACAAGGCCCTGGGGCTCCTGTGTGGGAAGGAGGCCGCAGCCTPteropus_alecto              AAGTAATGACAAAGCCCTGGGACTCCTGTGTGGGAAGGACGCCAAAGACTArtibeus_jamaicensis         AAGTAACGACAAAGCCCTGGGACTCCTGTGCGGGAAGGAGGCTGAGGCCTCynopterus_sphinx            AAGTAATGACAAAGCCTTGGGACTCCTGTGTGGGAAGGATGCCGAAGCCTDesmodus_rotundus            AAGTAACGACAAAGCCCTGGGACTCTTGTGCGGGAAGGACGCCGAGGCCT                              ** ** ***** *** **** *** **** ******** **    * **Epomops_beuttikoferi         GCAATGCTACCAACTGGATTGAGTACATGTTCAATAAGGACAATGGCCAGEidolon_helvum               GCAATGCTACCAACTGGATTGAGTACATGTTCAATAAGGACAATGGCCAGHypsignathus_monstrosus      GCAATGCTACCAACTGGATTGAGTACATGTTCAATAAGGACAATGGCCAGRousettus_aegyptiacus        GCAATGCTACCAACTGGATTGAGTACATGTTCAATAAGGACAATGGCCAGMyotis_lucifugus             GCAACGCCACCAACTGGATCGAGTACATGTTCAACAAGGACAACGGCCAGMyotis_brandtii              GCAACGCCACCAACTGGATCGAGTACATGTTCAACAAGGACAACGGCCAGPteropus_alecto              GCAATGCTACCAACTGGATTGAGTACATGTTCAATAAGGACAATGGCCAGArtibeus_jamaicensis         GCAACGCTACCAACTGGATCCAGTACATGTTCAACAAGGACAACGGCCAGCynopterus_sphinx            GCAATGCTACCAACTGGATCGAGTACATGTTCAATAAGGACAATGGCCAGDesmodus_rotundus            GCAACGCCACCAACTGGATCGAGTACATGTTCAATAAGGACAACGGCCAG                             **** ** ***********  ************* ******** ******Epomops_beuttikoferi         GCACCTTTCACCATCATACCCATTTTTTCAGATCTTCCAGCCCATGGGATEidolon_helvum               GCACCTTTCACCATCATACCCATTTTTTCAGATCTTCCAACCCATGGGATHypsignathus_monstrosus      GCACCTTTCACCATCATACCCATTTTTTCAGATCTTCCAGCCCATGGGATRousettus_aegyptiacus        GCACCTTTCACCATCATACCCATTTTTTCAGATCTTCCAGCCCATGGGATMyotis_lucifugus             GCCCCCTTCACCATCACTCCCGTATTCTCAGACCTCCCGGCCCACGGGATMyotis_brandtii              GCCCCCTTCACCATCACTCCCGTCTTCTCAGACCTCCCGGCCCACGGGATPteropus_alecto              GCACCTTTCACTATCACACCCATTTTTTCAGATCTTCCAGCCCATGGGATArtibeus_jamaicensis         GCACCTTTCACCATCACGCCCATTTTTTCAGATCTCCCAACCCAGGGGATCynopterus_sphinx            GCACCTTTCACCATCACACCCATTTTTTCAGATCTACCAACCCACGGGATDesmodus_rotundus            GCGCCTTTCACCATCACGCCCATTTTTTCAGATCTCCCGACCCGAGGGAT                             ** ** ***** ****  *** * ** ***** ** **  ***  *****Epomops_beuttikoferi         GGAGCCCATGAACAATGCCACCAAGGGCTGTGATGAGTCTGTGGATGAGGEidolon_helvum               GGAGCCCATGAACAATGCCACCAAGGGCTGTGATGAGTCTGTGGATGAGGHypsignathus_monstrosus      GGAGCCCATGAACAATGCCACCAAGGGCTGTGATGAGTCTGTGGATGAGGRousettus_aegyptiacus        GGAGCCCATGAACAATGCCACCAAGGGCTGTGATGAGTCTGTGGATGAGGMyotis_lucifugus             GGAGCCGATGAACAACGCCACCAAGGGCTGTGCCGAGCCCGTGGACGAGGMyotis_brandtii              GGAGCCGATGAACAACGCCACCAAGGGCTGTGCCGAGCCCGTGGACGAGGPteropus_alecto              GAAGCCCATGAACAATGCTACCAAGGGCTGTGACGAGTCTGTGGATGAGGArtibeus_jamaicensis         GGAGCCGATGAACAACGCCACCAAGGGCTGTGCCGAGGCTGTGGACGAGGCynopterus_sphinx            GGAGCCCATGAACAATGCCACCAAGAGCTGTGATGAGTCTGTGGATGAGGDesmodus_rotundus            GGAGCCCATGAACAACGCCACCAAGGGCTGTGCCGAGGCTGTGGACGAGG                             * **** ******** ** ****** ******  *** * ***** ****Epomops_beuttikoferi         TCACGGGGCCATGCAGCTGCCAGGACTGCTCGGCTGTTTGTGGCCCCAAGEidolon_helvum               TCACGGGGCCGTGCAGCTGCCAGGACTGCTCGGCTGTTTGTGGCCCCAAGHypsignathus_monstrosus      TCACGGGGCCATGCAGCTGCCAGGACTGCTCGGCTGTTTGTGGCCCCAAGRousettus_aegyptiacus        TCACGGGGCCATGCAGCTGCCAGGACTGTTCTGCTGTTTGTGGCCCCAAGMyotis_lucifugus             TCACCGGGCCCTGCAGCTGCCAGGACTGCTCGGCCATGTGCGGCCCCCGAMyotis_brandtii              TCACCGGGCCCTGCAGCTGCCAGGACTGCTCGGCCGTGTGCGGCCCCCGCPteropus_alecto              TCACGGGGCCGTGCAGCTGCCAGGATTGCTCAGCCGTTTGTGGCCCCAAGArtibeus_jamaicensis         TCACTGGGCCCTGCAGCTGCCAGGACTGCTCCGCTGTGTGCGGCCCCAAGCynopterus_sphinx            TAACGGGGCCATGCAGCTGCCAAGACTGCTCATCTGTTTGTGGCCCCAAGDesmodus_rotundus            TCACCGGGCCCTGCAGCTGCCAGGACTGCTCCGCCGTGTGCGGCCCCAGG                             * ** ***** *********** ** ** **  *  * ** ******   Epomops_beuttikoferi         CCCAAGCCACCGCCCCCTCCTGTTCCCTGGAGAATCTTGGGCTTGGACGCEidolon_helvum               CCCAAGCCACCGCCCCCTCCTGTTCCCTGGAGAATCTTGGGCTTGGATGCHypsignathus_monstrosus      CCCAAGCCACCGCCCCCTCCTGTTCCCTGGAGAATCTTGGGCTTGGACGCRousettus_aegyptiacus        CCCAAGCCACCGCCCCCTCCTGTTCCCTGGAGAATCTTGGGCTTGGATGCMyotis_lucifugus             CCCCAGCCGCCGCCCCCTCCCGTCCCCTGGAGAATCTGGGGCCTGGACGCMyotis_brandtii              CCCCAGCCGCCGCCCCCGCCTGTCCCCTGGAGAATCTGGGGCCTGGACGCPteropus_alecto              CCCAAGCCACCACCCCCTCCTGTTCCCTGGAGAATCTTGGGCTTGGACGCArtibeus_jamaicensis         CCCCAACCCCCGCCCCCTCCCCTCCCCTGGAGAATCTGGGGCCTGGATGCCynopterus_sphinx            CCCGAGCCACCATCCCCTCCTGTTCCCTGGAGAATCTTGGGCTTGGACGCDesmodus_rotundus            CCCCAGCCCCCGCCCCCTCCCCGTCCCTGGACAGTCTGGGGCCTGGACGC                             *** * ** **  **** **    ******* * *** **** **** **Epomops_beuttikoferi         CATGTATGTCATCATGTGGACCACCTACATGGCATTTTTACTTGTGTTTTEidolon_helvum               CATGTATGTCATCATGTGGACCACCTACATGGCATTTTTACTTGTGTTTTHypsignathus_monstrosus      CATGTATGTCATCATGTGGACCACCTACATGGCATTTTTACTTGTGTTTTRousettus_aegyptiacus        CATGTATGTCATCATGTGGACCACCTACATGGCATTTTTAATTGTGTTTTMyotis_lucifugus             CATGTACGTCGTCATGTGGGCCACCTACATGGCGTTTTTGCTCATGTTTTMyotis_brandtii              CATGTACGTCGTCATGTGGGCCACCTACATGGCGTTTTTGCTCATGTTTTPteropus_alecto              CATGTATGTCATCATGTGGACCACCTACATGGCATTTTTACTTGTGTTTTArtibeus_jamaicensis         CATGTATATCATCATGTGGCTCACCTACATGGCGTTTCTGCTTGTGTTTTCynopterus_sphinx            CATGTATGTCATCATGTGGACCACCTACATGGCATTTTTACTTGTGTTTTDesmodus_rotundus            CATGTATGTCATCATGTGGCTCACCTACACGGCGTTTCTGCTCGTGTTTT                             ******  ** ********  ******** *** *** *  *  ******Epomops_beuttikoferi         TTGGAGCATTTTTTGCTGTGTGGTGCTACAGGAAACGGTATTTTGTCTCCEidolon_helvum               TTGGAGCATTTTTTGTTGTGTGGTGCTACAGGAAACGGTATTTTGTCTCCHypsignathus_monstrosus      TTGGAGCATTTTTTGCTGTGTGGTGCTACAGGAAACGGTATTTTGTCTCCRousettus_aegyptiacus        TTGGAGCATTTTTTGCTGTGTGGTGCTACAGGAAACGGTATTTTGTCTCCMyotis_lucifugus             TTGGAGCCATTTTTGCCGTGTGGTGCTATAGAAAACGGTACTTGGTCTCCMyotis_brandtii              TTGGAGCCATTTTTGCCGTGTGGTGCTATAGAAAACGGTACTTGGTCTCCPteropus_alecto              TTGGAGCATTTTTTGCTGTGTGGTGCTACAGGAAACGGTATTTTGTCTCCArtibeus_jamaicensis         TTGGAGCATTTTTTGCCGTGTGGTGCTACAGAAAACGGTATTTTGTCTCCCynopterus_sphinx            TTGGAGCATTTTTTGCTGTATGGTGCTACAGGAAACGGTATTTTGTCTCCDesmodus_rotundus            TTGGAGCATTTTTTGCCGTGTGGTGCTACAGAAAACGGTATTTTGTCTCC                             *******  ******  ** ******** ** ******** ** ******Epomops_beuttikoferi         GAGTACACTCCCATTGATAGCAATATAGCTTTCTCAGTCAATGCCAGAGAEidolon_helvum               GAGTACACCCCCATTGATAGCAATATAGCTTTCTCTGTCAATGCCAGAGAHypsignathus_monstrosus      GAGTACACTCCCATTGATAGCAATATAGCTTTCTCAGTCAATGCCAGAGARousettus_aegyptiacus        GAGTACACTCCCATTGATAGCAATATAGCTTTCTCAGTCAACGCCAGAGAMyotis_lucifugus             GAGTACACCCCCATTGATAGCAGCATAGCTTTCCCGATGAGTGTCAGCGAMyotis_brandtii              GAGTACACCCCCATTGATAGCAGCATAGCTTTCCCGATGAGTGTCAGCGAPteropus_alecto              GAGTACACCCCCATTGATAGCAATATAGCTTTCTCTGTCAATGCCAGAGAArtibeus_jamaicensis         GAGTACACCCCCATCGATAGCAATATTGCCTTCCCTGTATTTGCCCGTGACynopterus_sphinx            GAGTACACCCCCATTGATAGCAATATAGCTTTCTCTGTCAATGCCAGAGADesmodus_rotundus            GAGTACACCCCCATCGACAGCAATATCGCTTTCCCTGTATTTGCCCGTGA                             ******** ***** ** ****  ** ** *** *  *    * * * **Epomops_beuttikoferi         CAAAGGGGAGGCATCCTGCTGCGACCCTCTTGGTGCAGCATTTGAGGGCTEidolon_helvum               CAAAGGGGAGGCATCCTGCTGCGACCCACTTGGTGCAGCATTTGAGGGCTHypsignathus_monstrosus      CAAAGGGGAGGCATCCTGCTGCGACCCTCTTGGTGCAGCATTTGAGGGCTRousettus_aegyptiacus        CAAAGGGGAGGCATCCTGCTGCGACCCTCTTGGTGCAGCATTTGAGGGCTMyotis_lucifugus             CACAGGGGAGGCCTCCTGCTGCGACCCGCTTGGCGCCGCATTCGAGGGCTMyotis_brandtii              CACAGGGGAGGCCTCCTGCTGCGACCCGCTTGGTGCCGCATTCGAGGGCTPteropus_alecto              CAAAGGGGAGGCATCCTGCTGCGACCCACTCGGTGCAGCATTTGAGGGCTArtibeus_jamaicensis         CAAAGGGGAGGCGCCTTGCTGCAGCCCGCTCGGTGAAGTGTTTGAGGGCTCynopterus_sphinx            CAAAGGGGAGGCATCCTGCTGCGACCCACTTGGTGCAGCATTTGAGGGCTDesmodus_rotundus            CAAAGGGGAGGCGCCATGCTGCAGCCCGCTTGGTGAAGTGTTTGAGGGCT                             ** *********  * ******  *** ** ** *  *  ** *******Epomops_beuttikoferi         GTTTGAGGCGGCTCTTCACACAGTGGGGTTCCTTCTGTGTCCGAAACCCTEidolon_helvum               GTTTGAGGCGGCTCTTCACACAGTGGGGTTCCTTCTGTGTCCGAAACCCTHypsignathus_monstrosus      GTTTGAGGCGGCTCTTCACACAGTGGGGTTCCTTCTGTGTCCGAAACCCTRousettus_aegyptiacus        GTTTGAGGCGGCTCTTCACACAGTGGGGTTCCTTCTGTGTCCGAAACCCTMyotis_lucifugus             GCTTGAGGCGGCTCTTCACCCGGTGGGGCTCCTTCTGTGTCCGAAACCCCMyotis_brandtii              GCTTGAGGCGGCTCTTCACCCGGTGGGGCTCCTTCTGTGTCCGAAACCCCPteropus_alecto              GTTTGAGGCGGCTCTTCACACAGTGGGGCTCTTTCTGTGTCCGAAACCCTArtibeus_jamaicensis         GTTTGAGGCAGCTTTTCACACAGTGGGGCTCCTTCTGTGTCCAAAACCCCCynopterus_sphinx            GTTTGAGGTGGCTCTTCACACAGTGGGGTTCCTTCTGTGTCCGAAACCCTDesmodus_rotundus            GTTTGAGGCAGCTTTTCACGTGGTGGGGCTCCTTCTGTGTCCAAAACCCC                             * ******  *** *****   ****** ** ********** ****** Epomops_beuttikoferi         GGCTGCGTTATTTTCTTCTCCCTGGTCTTTATCGCCACATGTTCTTCGGGEidolon_helvum               GGCTGCGTTATTTTCTTCTCCCTGGTCTTTATTGCCACATGTTCTTCGGGHypsignathus_monstrosus      GGCTGCGTTATTTTCTTCTCCCTGGTCTTTATCGCCACATGTTCTTCGGGRousettus_aegyptiacus        GGCTGCGTTATTTTCTTCTCCCTGGTCTTTATCGCCACATGTTCTTCGGGMyotis_lucifugus             GGCTGTGTCATTTTCTCCTCCCTGGCCTTCATCGCCGCCTGCTCCTCGGGMyotis_brandtii              GGCTGTGTCATTTTCTCCTCCCTGGCCTTCATCGTCGCCTGCTCCTCGGGPteropus_alecto              GGCTGCGTTATTTTCTTCTCCCTGGTCTTTATCGCCACATGTTCTTCGGGArtibeus_jamaicensis         GGCTGCGTCATTTTCTTCTCCTTGGTCTTCATTGCTGCCTGTTCTTTAGGCynopterus_sphinx            GGCTGCGTTATTTTCTTCTCCCTGGTCTTTATCGCCATGTGTTCTTCGGGDesmodus_rotundus            GGCTGCGTCCTTTTCTTCTCCTTGGTCTTCATTGCTGCCTGTTCTTTAGG                             ***** **  ****** **** *** *** ** *     ** ** *  **Epomops_beuttikoferi         CCTGGTGTTTGTCCAGGTTACAACCAATCCAGTTGACCTCTGGTCAGCCCEidolon_helvum               CCTGGTGTTTGTCCAGGTCACAACCAATCCAGTTGACCTCTGGTCAGCCCHypsignathus_monstrosus      CCTGGTGTTTGTCCAGGTTACAACCAATCCAGTTGACCTCTGGTCAGCCCRousettus_aegyptiacus        CCTGGTGTTTGTCCAGGTCACAACCAATCCAGTTGACCTCTGGTCAGCCCMyotis_lucifugus             CCTGGTGTTCGTCCGCATCACCACCAGCCCCGTGGACCTCTGGTCGGCCCMyotis_brandtii              CCTGGTGTTCGTCCGCGTCACCACCAGCCCCGTGGACCTCTGGTCGGCCCPteropus_alecto              CCTGGTGTTTGTCCAGGTCACAACCAATCCAGTTCACCTCTGGTCAGCCCArtibeus_jamaicensis         CCTGATGTTTGTCCACGTCACGACCAATCCAGTCGACCTCTGGTCAGCTCCynopterus_sphinx            CCTGGTGTTTGTTCAGGTCACAACCAATCCAGTAGACCTCTGGTCAGCCCDesmodus_rotundus            CCTGATGTTTGTCCAAGTCACGACCAATCCAGTTGACCTCTGGTCAGCCC                             **** **** ** *   * ** ****  ** **  ********** ** *Epomops_beuttikoferi         CCAGCAGCCAAGCGCGCCTGGAGAAAGATTACTTTGATACCCACTTTGGGEidolon_helvum               CCAGCAGCCAGGCGCGCCTGGAAAAAGAGTACTTTGATACCCACTTTGGGHypsignathus_monstrosus      CCAGCAGCCAAGCGCGCCTGGAGAAAGATTACTTTGATACCCACTTTGGGRousettus_aegyptiacus        CCAACAGCCAGGCGCGTCTGGAAAAAGAGTACTTTGATACCCACTTTGGGMyotis_lucifugus             CCAGCAGCCAGGCGCGCCTGGAGAAGGAGTACTTCGACACCCACTTCGGGMyotis_brandtii              CCAGCAGCCAGGCGCGCCTGGAGAAGGAGTACTTCGACACCCACTTCGGGPteropus_alecto              CCAGCAGCCAGGCGCGCCTAGAAAAAGAGTACTTTGATACCCACTTTGGGArtibeus_jamaicensis         CCAGCAGCCAGGCACGCCTGGAAAAAGAGTACTTTGACACCCACTTTGGGCynopterus_sphinx            CCAGCAGCCAGGCACGCCTGGAAAAAGAATACTTTGACACCCACTTTGGGDesmodus_rotundus            CCAGCAGCCAGGCACGCCTGGAAAAAGAGTACTTTGACACCCACTTCGGG                             *** ****** ** ** ** ** ** ** ***** ** ******** ***Epomops_beuttikoferi         CCTTTCTTTCGCCTGGAGCAACTCATCATCCGGGCCCCCCACACCGCCATEidolon_helvum               CCTTTCTTTCGCACGGAGCAGCTCATCATCCAGGCCCCCCACACCGCCACHypsignathus_monstrosus      CCTTTCTTTCGCATGGAGCAACTCATCATCCGGGCCCCCCACACCGCCATRousettus_aegyptiacus        CCTTTCTTTCGCGTGGAGCAACTCATCATCCAGGCCCCCCACACCGCCATMyotis_lucifugus             CCTTTCTTCCGCACGGAGCAGCTCATCATCCAGGCGCCGCACACCGCCGCMyotis_brandtii              CCTTTCTTCCGCACGGAGCAGCTCATCATCCAGGCGCCGCACACCGCCGTPteropus_alecto              CCTTTCTTTCGCACGGAGCAGCTCATCATCCGGGCCCCCCACACCGCCACArtibeus_jamaicensis         CCTTTTTACCGCATAGAGCAGCTTATTATCCGAGCCCCCCACAGCCACACCynopterus_sphinx            CCTTTCTTTCGCACGGAGCAGCTCATCATCCGGGCCCCCCACACTGCCACDesmodus_rotundus            CCTTTTTTCCGCACAGAGCAGCTTATTATCCGGGCCCCCCACACCCCCAC                             ***** *  ***   ***** ** ** ****  ** ** ****    *  Epomops_beuttikoferi         GCACACTTACCAGCCGTACCCCGCAGGGTCTGATGTGCCCTTTGGACCTCEidolon_helvum               GCACACTTACCAGCCATACCCCGCAGGGTCTGATGTGCCCTTTGGACCTCHypsignathus_monstrosus      GCACACTTACCAGCCGTACCCCGCAGGGTCTGATGTGCCCTTTGGACCTCRousettus_aegyptiacus        GCACACTTACCAGCCATACCCCGCAGGATCTGATGTGCCCTTTGGACCTCMyotis_lucifugus             GCACACTTACGAGCCGTACCCCTCGGGGTCCGACGTGCCCTTCGGACCTCMyotis_brandtii              GCACACTTACGAGCCATACCCCTCGGGGTCCGACGTGCCCTTCGGACCTCPteropus_alecto              ACACACTTACCAGCCATACCCCACAGAGACCGATGTGCCCTTTGGACCTCArtibeus_jamaicensis         ACACACCTACCAGCCATACCCTTCCGGATCTGATGTGCCCTTTGGACCTCCynopterus_sphinx            ACACACTTATCAGCCATACCCTGCAGGATCCGATGTGCCCTTTGGACCTCDesmodus_rotundus            ACACACCTACCAGCCGTACCCCTCCGGATCCGACGTGCCCTTTGGACCTC                              ***** **  **** *****  * *   * ** ******** *******Epomops_beuttikoferi         CGCTGGACATAGAAATTTTGCACCAGGTTCTTGACTTACAGACAGCCATCEidolon_helvum               CGCTGGACATAGAAATTTTGCACCAGGTTCTTGACTTACAAACAGCCATCHypsignathus_monstrosus      CGCTGGACATAGAAATTTTGCACCAGGTTCTTGACTTACAGACAGCCATCRousettus_aegyptiacus        CGCTGAACATAGAAATTTTGCACCAGGTTCTTGACTTACAAACAGCCATCMyotis_lucifugus             CGCTCGACAAAGGGATCCTGCACCAGGTTCTTGACTTACAAACAGCCATTMyotis_brandtii              CGCTCGACAAAGGGATTCTGCACCAGGTTCTTGACTTACAAACAGCCATTPteropus_alecto              CACTGGACATAGAAATTTTGCACCAGGTTCTTGACTTACAAACAGCCATCArtibeus_jamaicensis         CGCTTAACATAGAGATTTTGCACCAGGTTCTTGACTTACAAACAGCCATCCynopterus_sphinx            CGCTGGACATAGAAATTTTGCACCAGGTTCTTGACCTACAAACAGCCATTDesmodus_rotundus            CGCTTAACATAGAGATTTTGCACCAGGTTCTTGACTTACAAACAGCCATC                             * **  *** **  **  ***************** **** ******** Epomops_beuttikoferi         GAAAACATTAGTGTGTCTTACAACAACGAGACCGTGTCACTTCAAGACATEidolon_helvum               GAAAACATTAGTGTGTCTTATAACAACGAGACTGTGTCACTTCAAGACATHypsignathus_monstrosus      GAAAACATTAGTGTGTCTTACAACAACGAGACCGTGTCACTTCAAGACATRousettus_aegyptiacus        GAAAACATTAGTGTGTCTTATAACAACGAGACCGTGTCACTTCAAGACATMyotis_lucifugus             GAACACATCACTGCATCTCACAACAACGAGACGGTGACCCTCCAGGACATMyotis_brandtii              GAACACATCACTGCATCTTACAACAACGAGACCGTGACCCTCCAGGACATPteropus_alecto              GAAAACATTAGTGTGTCTTATAACAACGAGACCGTGTCACTTCAAGACATArtibeus_jamaicensis         GAAAACATTACTGCATCTTACAACAACGAGACTGTGACACTTCAAGACATCynopterus_sphinx            GAAAACATTAGTGTGTCTTATAACAACGAGACTGTGTCACTTCAAGATATDesmodus_rotundus            GAAAGCATTACTGCATCTTATAACAACGAGACTGTGACACTTCAAGACAT                             ***  *** * **  *** * *********** *** * ** ** ** **Epomops_beuttikoferi         CTGCTTGGCCCCTCTCTCACCATATAACAAGAACTGTACCATTATGAGTGEidolon_helvum               CTGCTTGGCCCCTCTCTCACCGTATAACAAGAACTGTACCATTTTGAGTGHypsignathus_monstrosus      CTGCTTGGCCCCTCTCTCACCATATAACAAGAACTGTACCATTATGAGTGRousettus_aegyptiacus        CTGCTTGGCCCCTCTCTCACCATATAACAAGAACTGTACCATTATGAGTGMyotis_lucifugus             CTGCTTGGCCCCGCTCTCGCCGTATAACAAGAACTGCACCATCCTGAGTGMyotis_brandtii              CTGCTTGGCCCCGCTCTCCCCGTATAACAAGAACTGCACCATCCTGAGTGPteropus_alecto              CTGCTTGGCCCCTCTATCACCATATAACAAGAACTGTACCATTATGAGTGArtibeus_jamaicensis         CTGCTTGGCCCCGCTCTCACCATATAACCAAAACTGCACCATCATGAGTGCynopterus_sphinx            CTGCTTGGCTCCTCTCTCACCATATAACAAGAACTGTACCATTATGAGTGDesmodus_rotundus            CTGCTTGGCCCCTCTCTCACCATATAACAAAAACTGCACCATTCTGAGTG                             ********* ** ** ** ** ****** * ***** *****  ******Epomops_beuttikoferi         TGTTGAACTACTTCCAGAACAGCCACTCGATGCTGGACCACAAAATAGGGEidolon_helvum               TGTTGAATTACTTCCAGAACAATCACTTGACGCTGGACCACAAAATAGGGHypsignathus_monstrosus      TGTTGAACTACTTCCAGAACAGCCACTCGATGCTGGACCACAAAATAGGGRousettus_aegyptiacus        TGTTGAATTACTTCCAGAACAGTCACTCGATGCTGGACCACAAAATAGGGMyotis_lucifugus             TGTTAAATTACTTCCAGAACAGCCACTCCGTGCTGGACCACGAGATAGGGMyotis_brandtii              TGTTAAATTACTTCCAGAACAGCCACTCCATGCTGGACCACGAGATAGGGPteropus_alecto              TGTTGAATTACTTCCAGAACAGTCACTCGACGCTGGACCACAAAATAGGGArtibeus_jamaicensis         TGCTGAACTACTTCCAGAACAGCCACTCCATGCTGGACCACAAAATAGGGCynopterus_sphinx            TGTTGAATTACTTCCAGAACAGTCATTCAGTGCTGGACCACAAGATCGGGDesmodus_rotundus            TGTTGAATTACTTCCAGAACAGCCACTCCCTGCTGGACCACAAAATAGGG                             ** * ** *************  ** *    ********** * ** ***Epomops_beuttikoferi         GATGACTTCTTTGTGTATGCGGATTACCACACGCACTTTCTGTACTGTGTEidolon_helvum               GATTTCTTCTTTACGTATGCGGATTACCACACGCACTTTCTGTACTGTGTHypsignathus_monstrosus      GATGACTTCTTTGTGTATGCGGATTACCACACGCACTTTCTGTACTGTGTRousettus_aegyptiacus        GATGACTTCTTTGTGTATGCGGATTACCACACGCACTTTCTGTACTGTGTMyotis_lucifugus             GACGACTTCTATACCTACGCGGATTACCACACGCACCTGCTGTACTGTGTMyotis_brandtii              GACGACTTCTATACCTATGCGGATTACCACACGCACCTGCTGTACTGTGTPteropus_alecto              GATGACTTCTACGTGTATGCGGATTACCACACGCACTTTCTGTACTGTGTArtibeus_jamaicensis         GACCCCTTCTATGTGTACGCGGATTACCACACGCACTTTCTGTACTGTGTCynopterus_sphinx            GATGACTTCTTTGTGTATGCGGATTACCACACACACTTTCTGTACTGTGTDesmodus_rotundus            GACGCCTTCTACGTGTACGCGGATTACCACACGCACTTTCTGTACTGTGT                             **   *****     ** ************** *** * ***********Epomops_beuttikoferi         ACGGGCTCCTGCCTCTCTGAATGATACCAGTTTGCTCCATGATCCTTGCCEidolon_helvum               ACGGGCTCCTGCCTCTCTGAATGATACCAGTTTGCTCCATGATCCTTGCCHypsignathus_monstrosus      ACGGGCTCCTGCCTCTCTGAATGATACCAGTTTGCTCCATGATCCTTGCCRousettus_aegyptiacus        ACGGGCTCCCGCCTCTCTGAATGATACCAGTTTGCTCCATGATCCTTGCCMyotis_lucifugus             CCGGGCTCCTGCCTCGCTGAACGACACCAGCCTGCTGCACGACCCCTGCCMyotis_brandtii              CCGGGCTCCTGCCTCGCTGAACGACACCAGCCTGCTGCACGACCCCTGCCPteropus_alecto              ACGGGCTCCTGCCTCTCTGAATGATACCAGTTTGCTCCATGATCCTTGCCArtibeus_jamaicensis         ACGGGCCCCCGCCTCTCTGAATGATACCAGTGTGCTTCACGACCCCTGTCCynopterus_sphinx            ACGGGCTCCTGCCTCTCTGAATGATACCAGTTTGCTTCATGATCCTTGCCDesmodus_rotundus            ACGGGCTCCCGCCTCTCTGAATGACACCAGTGTGCTCCACGATCCTTGTC                              ***** ** ***** ***** ** *****  **** ** ** ** ** *Epomops_beuttikoferi         TGGGTACATTTGGTGGACCAGTATTCCCATGGCTCGTGTTAGGGGGCTATEidolon_helvum               TGGGTACATTTGGTGGACCAATATTCCCATGGCTCGTGTTAGGGGGCTATHypsignathus_monstrosus      TGGGTACATTTGGTGGACCAGTATTCCCATGGCTCGTGTTAGGGGGCTATRousettus_aegyptiacus        TGGGTACATTTGGGGGGCCAGTATTCCCATGGCTCGTGTTAGGGGGCTATMyotis_lucifugus             TGGGGACGTTTGGGGGGCCTGTGTTCCCGTGGCTGGTGCTGGGAGGCTATMyotis_brandtii              TGGGGACCTTTGGGGGGCCTGTGTTCCCGTGGCTGGTGCTGGGAGGCTATPteropus_alecto              TGGGGACATTTGGTGGACCAGTATTCCCATGGCTCGTGTTAGGGGGCTATArtibeus_jamaicensis         TGGGCACGTTTGGTGGGCCCGTGTTCCCCTGGCTCGTGTTAGGTGGCTATCynopterus_sphinx            TGGGTACGTTTGGTGGACCAGTATTCCCATGGCTCGTGTTAGGCGGCTATDesmodus_rotundus            TGGGTACGTTCGGGGGACCGGTGTTCCCCTGGCTTGTGTTAGGCGGCTAT                             **** ** ** ** ** **  * ***** ***** *** * ** ******Epomops_beuttikoferi         GACGGTCAAAACTACAATAACGCCACAGCCCTTGTAATTACCTTTCCTGTEidolon_helvum               GATGATCAAAACTACAATAATGCCACAGCCCTTGTGATTACTTTTCCTGTHypsignathus_monstrosus      GACGGTCAAAACTACAATAACGCCACAGCCCTTGTAATTACCTTTCCTGTRousettus_aegyptiacus        GATGATCAAAACTACAATAATGCCACAGCCCTTGTAATTACCTTTCCTGTMyotis_lucifugus             GACGATCAAAACTACAATAACGCCACCGCCCTGGTGATGACCTTTCCTGTMyotis_brandtii              GACGATCAAAACTACAATAACGCCACCGCCCTGGTGATGACCTTTCCTGTPteropus_alecto              GATGATCAAAACTACAGTAATGCCACAGCCCTTGTGATTACCTTACTTGTArtibeus_jamaicensis         GATGATCAAAACTACAATAACGCCACAGCCCTCGTGATCACCTTCCCTGTCynopterus_sphinx            GATGATCAAAACTACAATAATGCCACAGCCCTTGTGATTACCCTTCCTGTDesmodus_rotundus            GATGATCAAAACTACAATAACGCCACAGCCCTCGTGATTACCTTTCCTGT                             ** * *********** *** ***** ***** ** ** **  * * ***Epomops_beuttikoferi         CAGTAACTATTATAATGATACAGAGAAGCTCCAGAGAGCCCAGGCCTGGGEidolon_helvum               CAATAATTATTATAATGATACAGAGAAGCTCCAGAGGGCCCAGGCCTGGGHypsignathus_monstrosus      CAGTAACTATTATAATGATACAGAGAAGCTCCAGAGAGCCCAGGCCTGGGRousettus_aegyptiacus        CAATAACTATTATAATGATACAGAGAAGCTCCAGAGGGCCCAGGTCTGGGMyotis_lucifugus             CAATAACTACTACAACGACACGGAGAAGCTCCAGCGGGCCCAGGCCTGGGMyotis_brandtii              CAATAATTACTACAACGACACGGAGAAGCTCCAGCGGGCCCAGGCCTGGGPteropus_alecto              CAATAATTATTATAATAATACCGAGAAGCTCCAGAGGGCCCAGGCCTGGGArtibeus_jamaicensis         CAACAATTACTACAATGACACGGAGAAGCTCCAGAGGGCCCAGGCCTGGGCynopterus_sphinx            CAATAATTATTATAATGATACAGAGAAGCTCCAGAGGGCCCAGGCCTGGGDesmodus_rotundus            CAATAATTACTACAATGATACTGAGAAGCTCCAGAGGGCCCAGGCCTGGG                             **  ** ** ** **  * ** ************ * ******* *****Epomops_beuttikoferi         AACAAGAGTTTATTAATTTTGTGAAGAACTACAAGAATCCAAATCTGACCEidolon_helvum               AACGAGAGTTTATTAATTTCGTGAAAAACTACAAGAATCCAAATCTGACCHypsignathus_monstrosus      AACAAGAGTTTATTAATTTTGTGAAGAACTACAAGAATCCAAATCTGACCRousettus_aegyptiacus        AACGAGAGTTTATTAATTTTGTGAAAAACTATAAGAATCCAAATCTTACCMyotis_lucifugus             AAAGAGAGTTTATTAACTTTGTGAAGAACTACAACAACCCAAATCTCACCMyotis_brandtii              AAAGAGAGTTTATTAACTTTGTGAAAAACTACAAGAACCCAAATCTCACCPteropus_alecto              AACGAGAATTTATTAATTTCGTGAAAAACTACAAGAATCCAAATCTGACCArtibeus_jamaicensis         AAAGAGAGTTTATTAATTTTATGAAAAACTACGAGAATCCAAATCTCACCCynopterus_sphinx            AACGAGAGTTTATTAATTTCGTGAAAAACTATAAGAATCCAAATCTGACCDesmodus_rotundus            AAAAAGAGTTTACTAATTTTGTGAAAAACTACAAGAATCCAAATCTTACC                             **  *** **** *** **  **** *****  * ** ******** ***Epomops_beuttikoferi         ATTTCTTTCACTGCTGAACGAAGTATTGAAGATGAACTAAATCGTGAAAGEidolon_helvum               ATTTCTTTCACTGCTGAACGAAGTATTGAAGATGAACTAAATCGTGAAAGHypsignathus_monstrosus      ATTTCTTTCACTGCTGAACGAAGTATTGAAGATGAACTAAATCGTGAAAGRousettus_aegyptiacus        ATTTCTTTCACTGCCGAAAGAAGTATTGAAGATGAACTAAATCGTGAAAGMyotis_lucifugus             ATTTCCTTCACTGCTGAGCGAAGTATTGAAGATGAACTGAACCGTGAGAGMyotis_brandtii              ATTTCCTTCACTGCTGAGCGAAGTATTGAAGATGAACTAAACCGTGAGAGPteropus_alecto              ATTTCTTTCACTGCTGAACGAAGCATTGAAGATGAACTAAATCGTGAAAGArtibeus_jamaicensis         ATTTCTTTCACTGCTGAACGAAGTATCGAAGATGAACTGAACCGTGAAAGCynopterus_sphinx            ATTTCTTTCACTGCTGAACGAAGTATTGAAGATGAACTAAATCGTGAAAGDesmodus_rotundus            ATTTCTTTCACTGCTGAGCGAAGCATCGAAGACGAACTGAACCGTGAAAG                             ***** ******** **  **** ** ***** ***** ** ***** **Epomops_beuttikoferi         TAACAGTGATATCTTCACCATTGTAATCAGCTATGCTGTCATGTTTCTGTEidolon_helvum               TAACAGTGATATCTTCACTGTTGTAATCAGCTATGCTGTCATGTTTCTGTHypsignathus_monstrosus      TAACAGTGATATCTTCACCATTGTAATCAGCTATGCTGTCATGTTTCTGTRousettus_aegyptiacus        TAACAGTGATATCTTCACTGTTGTAATCAGCTATGCCGTCATGTTTCTGTMyotis_lucifugus             TAACAGTGACATCTCCACCGTGGTGACCAGCTATGCCGTCATGTTTCTGTMyotis_brandtii              TAACAGTGACATCTCCACCGTGGTGACCAGCTATGCCGTCATGTTTCTGTPteropus_alecto              TAACAGTGATATCTTCACTGTTGTAATCAGCTATGCCGTCATGTTTCTGTArtibeus_jamaicensis         TAGCGGTGACGTCTTCACTGTGATCATCAGCTACGCGGTCATGTTCCTGTCynopterus_sphinx            TAACAGTGATATCTTCACTGTTGTAATCAGCTATGCCGTCATGTTTGTGTDesmodus_rotundus            CAACAGTGACGTCTTCACGGTGATAATCAGCTACGCGGTCATGTTCCTGT                              * * ****  *** ***  *  * * ****** ** ********  ***Epomops_beuttikoferi         ATATTTCCATAGCCTTGGGGCACATCAAAAGCTGTAGCAGGCTTTTGGTAEidolon_helvum               ATATTTCCATAGCCTTGGGGCACATCAAAAGCTGTAGCAGGCTTTTGGTAHypsignathus_monstrosus      ATATTTCCATAGCCTTGGGGCACATCAAAAGCTGTAGCAGGCTTTTGGTARousettus_aegyptiacus        ATATTTCCATAGCCTTGGGGCACATCAAAAGCTGTAGCAGACTTTTGGTAMyotis_lucifugus             ACATTTCCATCGCCTTGGGGCACATCCAGAGCTGCAGCAGGCTTCTGGTGMyotis_brandtii              ACATTTCCATCGCCTTGGGGCACATCCAGAGCTGCAGCAGGCTTCTGGTGPteropus_alecto              ATATTTCCATAGCCTTGGGGCACATCAAAAGCTGTAGCAGGCTTTTGGTAArtibeus_jamaicensis         ATATTTCCATAGCCTTGGGGCACATCAAAAGCTGCAGCCGGCTTCTGGTGCynopterus_sphinx            ATATTTCCATAGCCTTGGGGCACATCAAAAGCTGTAGCAGGCTTTTGGTADesmodus_rotundus            ATATTTCCATAGCCTTGGGGCACATCAAAAGCTGCAGCCGGTTTCTGGTG                             * ******** *************** * ***** *** *  ** **** Epomops_beuttikoferi         GATTCTAAAATCTCCCTTGGCATCACGGGTATCCTCATCGTGTTAAGCTCEidolon_helvum               GATTCTAAAATCTCTCTCGGCATCACGGGTATCCTCATCGTGTTGAGCTCHypsignathus_monstrosus      GATTCTAAAATCTCCCTTGGCATCACGGGTATCCTCATCGTGTTAAGCTCRousettus_aegyptiacus        GATTCTAAAGTCTCCCTCGGCATCACGGGTATCCTCATCGTGTTGAGCTCMyotis_lucifugus             GATTCCAAGGTCTCCCTCGGCATCGCAGGCATCTTCATCGTGCTGAGCTCMyotis_brandtii              GATTCCAAGGTCTCCCTCGGCATCGCGGGCATCTTCATTGTGCTGAGCTCPteropus_alecto              GATTCTAAAATCTCCCTTGGCATCACGGGTATCTTCATCGTGTTGAGCTCArtibeus_jamaicensis         GACTCTAAGATCTCCCTGGGCATCGCGGGCATCCTCATTGTGTTGAGCTCCynopterus_sphinx            GATTCTAAAATCTCCCTCGGCATCACGGGTATCCTCATAGTGTTGAGCTCDesmodus_rotundus            GATTCTAAAATCTCCCTGGGCATCGCAGGCATCCTCATCGTGCTGAGCTC                             ** ** **  **** ** ****** * ** *** **** *** * *****Epomops_beuttikoferi         GGTGGCATGCTCGTTGGGCATCTTCAGCTATGCTGGGATCCCCCTCACCCEidolon_helvum               GGTGGCGTGCTCGTTGGGCATCTTCAGCTACTTTGGGATCCCCCTCACCCHypsignathus_monstrosus      GGTGGCATGCTCGTTGGGCATCTTCAGCTATGCTGGGATCCCCCTCACCCRousettus_aegyptiacus        GGTGGCGTGCTCGTTGGGCATCTTCAGCTACGCTGGGATCCCTCTCACCCMyotis_lucifugus             AGTGGCCTGCTCGCTGGGCATCTTCAGCTACGTTGGGGTCCCCCTCACCCMyotis_brandtii              AGTGGCCTGCTCGCTGGGCATCTTCAGCTACGTTGGGGTCCCCCTCACCCPteropus_alecto              GGTGGCGTGCTCGTTGGGCATCTTCAGCTACATCGGGATCCCCCTCACCCArtibeus_jamaicensis         GGTGGCGTGCTCACTGGGCATCTTCAGCTACTTTGGGATCCCCCTCACTCCynopterus_sphinx            CGTGGCGTGCTCATTGGGCATCTTCAGCTATGTTGGGATCCCCCTCACCCDesmodus_rotundus            AGTGGCGTGCTCATTGGGCATCTTCAGCTACTTTGGGGTCCCCCTCACCC                              ***** *****  ****************    *** **** ***** *Epomops_beuttikoferi         TCATTGTGATTGAAGTCATCCCGTTCCTGGTGCTGGCCGTTGGGGTGGACEidolon_helvum               TCATTGTGATTGAAGTCATCCCATTCCTGGTGCTGGCTGTTGGGGTGGACHypsignathus_monstrosus      TCATTGTGATTGAAGTCATCCCGTTCCTGGTGCTGGCCGTTGGGGTGGACRousettus_aegyptiacus        TCATTGTGATTGAAGTCATCCCATTCCTGGTGCTGGCCGTTGGGGTGGACMyotis_lucifugus             TCATTGTGATCGAGGTCATCCCCTTCCTGGTGCTGGCGGTCGGGGTGGACMyotis_brandtii              TCATTGTGATCGAGGTCATCCCCTTCCTGGTGCTGGCGGTCGGGGTGGACPteropus_alecto              TCATTGTGATTGAAGTCATCCCGTTCCTGGTGCTGGCCATTGGGGTGGACArtibeus_jamaicensis         TCATCGTGATTGAGGTCATCCCGTTCCTGGTGCTGGCTGTTGGGGTGGACCynopterus_sphinx            TCATTGTGATTGAAGTCATCCCATTCCTGGTGCTGGCCGTTGGGGTGGACDesmodus_rotundus            TCATCGTGATTGAAGTCATCCCATTCCTGGTGCTGGCTGTTGGGGTGGAC                             **** ***** ** ******** **************  * *********Epomops_beuttikoferi         AACATCTTCATTCTGGTCCAGACCTACCAGAGAGATGAACGTCTTCAAGGEidolon_helvum               AACATCTTCATTCTGGTCCAGACCTACCAGAGAGATGAACGTCTTCAAGGHypsignathus_monstrosus      AACATCTTCATTCTGGTCCAGACCTACCAGAGAGATGAACGTCTTCAAGGRousettus_aegyptiacus        AACATCTTCATTCTGGTCCAGACCTACCAGAGAGATGAACGTCTTCAAGGMyotis_lucifugus             AACATCTTCATTCTGGTCCAGACCTACCAGAGAGACGAACGCCTTGAAGGMyotis_brandtii              AACATCTTCATTCTGGTCCAGACCTACCAGAGAGACGAACGCCTTGAAGGPteropus_alecto              AACATCTTCATTCTGGTCCAGACCTACCAGAGAGATGAACGTCTTCAAGGArtibeus_jamaicensis         AACATCTTCATCCTGGTCCAGACCTACCAGAGAGATGAACGTCTTCAAGGCynopterus_sphinx            AACATCTTCATCCTGGTCCAGACCTACCAGAGAGATGAACGTCTTCAAGGDesmodus_rotundus            AACATCTTCATTCTGGTCCAGACCTACCAGAGAGACGAACGTCTTCAGGG                             *********** *********************** ***** *** * **Epomops_beuttikoferi         GGAAACCCTGGACCAGCAGCTGGGCAGGGTCCTTGGAGAAGTGGCTCCTAEidolon_helvum               GGAAACCCTGGACCAGCAGCTGGGCAGGGTCCTTGGAGAAGTGGCTCCTAHypsignathus_monstrosus      GGAAACCCTGGACCAGCAGCTGGGCAGGGTCCTTGGAGAAGTGGCTCCTARousettus_aegyptiacus        GGAAACCCTGGACCAGCAGCTGGGCAGGGTCCTTGGAGAAGTGGCTCCTAMyotis_lucifugus             GGAGACCCTGGACCAGCAGCTGGGCAGGGTCCTGGGAGAAGTGGCCCCCAMyotis_brandtii              GGAGACCCTGGACCAGCAGCTGGGCAGGGTCCTGGGAGAAGTGGCCCCCAPteropus_alecto              GGAAACCCTGGACCAGCAGCTGGGCAGGATCCTTGGAGAAGTGGCTCCTAArtibeus_jamaicensis         AGAAACCCTGGACCAGCAGCTGGGCAGGGTCCTTGGAGAAGTGGCTCCTACynopterus_sphinx            GGAAACCCTGGACCAGCAGCTGGGCAGGGTCCTTGGAGAAGTGGCTCCTADesmodus_rotundus            AGAAACCCTGGACCAGCAGCTGGGCAGGGTCCTTGGAGAAGTGGCTCCTA                              ** ************************ **** *********** ** *Epomops_beuttikoferi         GTATGTTCCTGTCATCCTTTTCAGAGACAGTAGCATTTTTCTTAGGAGCAEidolon_helvum               GTATGTTCCTGTCATCCTTTTCAGAGACAGTAGCATTTTTCTTAGGAGCAHypsignathus_monstrosus      GTATGTTCCTGTCATCCTTTTCAGAGACAGTAGCATTTTTCTTAGGAGCARousettus_aegyptiacus        GTATGTTCCTGTCATCCTTTTCAGAGACAGTAGCATTTTTCTTAGGAGCAMyotis_lucifugus             GCATGTTCCTGTCGTCCTTTTCAGAGACGGTGGCCTTTTTCTTAGGAGCCMyotis_brandtii              GCATGTTCCTGTCGTCCTTTTCAGAGACGGTGGCCTTTTTCTTAGGAGCCPteropus_alecto              GTATGCTCCTGTCATCCTTTTCAGAGACAGTAGCATTTTTCTTAGGAGCAArtibeus_jamaicensis         GTATGTTCCTGTCATCCTTTTCAGAGACGGTAGCATTTTTCTTAGGAGCCCynopterus_sphinx            GTATGTTCCTGTCATCCTTTTCAGAGACAGTAGCATTTTTCTTAGGAGCADesmodus_rotundus            GTATGTTCCTGTCGTCCTTTGCAGAGACAGTAGCATTTTTCTTAGGAGCC                             * *** ******* ****** ******* ** ** ************** Epomops_beuttikoferi         TTGTCAGTGATGCCAGCCGTTCATACTTTCTCTCTGTTTGCGGGAATGGCEidolon_helvum               TTGTCAATGATGCCAGCCGTTCATACTTTCTCTCTGTTTGCGGGAATGGCHypsignathus_monstrosus      TTGTCAGTGATGCCAGCCGTTCATACTTTCTCTCTGTTTGCGGGAATGGCRousettus_aegyptiacus        TTGTCAGTGATGCCGGCTGTTCATACTTTCTCTCTGTTTGCGGGAATGGCMyotis_lucifugus             CTGTCGGTGATGCCCGCCGTGCACACCTTCTCGCTGTTTGCCGGGATGGCMyotis_brandtii              CTGTCGGTGATGCCCGCCGTGCACACCTTCTCGCTGTTTGCCGGGATGGCPteropus_alecto              TTGTCAATGATGCCAGCCGTTCACACTTTCTCTCTGTTTGCGGGAATGGCArtibeus_jamaicensis         TTGTCCATGATGCCCGCCGTTCACACCTTCTCTCTGTTTGCCGGCTTGGCCynopterus_sphinx            TTGTCAATGATGCCAGCCGTTCATACTTTCTCTCTGTTTGCGGGAATGGCDesmodus_rotundus            TTGTCAGTGATGCCCGCCGTTCACACCTTCTCTCTGTTTGCCGGAATGGC                              ****  ******* ** ** ** ** ***** ******** **  ****Epomops_beuttikoferi         CGTCCTCATTGACTTCCTTCTTCAGATTACCTGTTTTGTGAGTCTCTTCGEidolon_helvum               AGTCCTCATTGACTTCCTTCTTCAGATTACCTGTTTTGTGAGTCTCTTGGHypsignathus_monstrosus      CGTCCTCATTGACTTCCTTCTTCAGATTACCTGTTTTGTGAGTCTCTTCGRousettus_aegyptiacus        AGTCCTCATTGACTTCCTTCTTCAGATTACCTGTTTTGTGAGTCTCTTCGMyotis_lucifugus             CGTCCTCATCGACTTCCTGCTCCAGATCACCTGCTTCGTGAGTCTCCTGGMyotis_brandtii              CGTCCTCATCGACTTCCTGCTCCAGATCACCTGCTTCGTGAGCCTCCTGGPteropus_alecto              AGTCTTCATTGACTTCCTTCTTCAGATTACCTGTTTTGTGAGTCTCTTGGArtibeus_jamaicensis         GGTCTTCATCGACTTCCTTCTTCAGATTACCTGTTTTGTGAGTCTCTTGGCynopterus_sphinx            AGTCCTCATTGACTTCCTTCTTCAGATTACCTGTTTTGTGAGTCTCTTGGDesmodus_rotundus            GGTCTTCATTGACTTCCTTCTTCAGATTACCTGTTTCGTGAGTCTCTTGG                              *** **** ******** ** ***** ***** ** ***** *** * *Epomops_beuttikoferi         GGTTAGACATTAAGCGTCAAGAGAAAAACCGGCTAGACATACTTTGCTGTEidolon_helvum               GGTTAGACATTAAGCGTCAAGAGAAAAACCGGCTAGACATACTTTGCTGTHypsignathus_monstrosus      GGTTAGACATTAAGCGTCAAGAGAAAAACCGGCTAGACATACTTTGCTGTRousettus_aegyptiacus        GGTTAGACATTAAGCGTCAAGAGAAAAACCGGCTAGACATACTTTGCTGTMyotis_lucifugus             GCTTGGACATTAAGCGTCAGGAGAACAACCGGCTGGACATCCTGTGCTGTMyotis_brandtii              GGTTGGACGTCAAGCGTCAGGAGAACAACCGGCTGGACATCCTGTGCTGTPteropus_alecto              GGTTAGACATTAAGCGTCAAGAGAAAAACCGGCTAGACGTGCTTTGCTGTArtibeus_jamaicensis         GCTTAGACATTAAGCGTCAAGAGAAAAACCGGCTGGACATCCTCTGCTGTCynopterus_sphinx            GGTTAGACATTAAGCGTCAAGAGAAAAACAGGCTAGACGTACTTTGCTGTDesmodus_rotundus            GCTTAGACATTAAGCGTCAAGAGAAAAACCGGCTGGACATCCTCTGCTGT                             * ** *** * ******** ***** *** **** *** * ** ******Epomops_beuttikoferi         GTCCGAGGTGCTGAAGATGGAACAGGCATCCAGGCCTCAGAGAGCTGCTTEidolon_helvum               GTCAGAGGTGCGGAAGATGGATCAGGCATCCAGGCTTCAGAGAGCTGCTTHypsignathus_monstrosus      GTCCGAGGTGCTGAAGATGGAACAGGCATCCAGGCCTCAGAGAGCTGCTTRousettus_aegyptiacus        GTCCGAGGTGCAGAAGATGGAACAGGCATCCAGGCCTCAGAGAGCTGCTTMyotis_lucifugus             GTCCGCGGCGGCGAGGATGGCGCCGGCCTCCAGGCCTCCGAGAGCTGCTTMyotis_brandtii              GTCCACGGCGGCGAGGATGGCGCCGGCCTCCAGGCCTCCGAGAGCTGCTTPteropus_alecto              GTCAGAGGTGCGGAAGATGGAACAGGCATCCAGGCCTCAGAGAGCTGCTTArtibeus_jamaicensis         GTCAGAGGCGCTGAAGATGGAACTGGCATCCAGGCCTCAGAGAGCTGCTTCynopterus_sphinx            GTCCAAGGTGCAGAAGATGGAACCGGCATCCAGGCCTCAGAGAGCTGCTTDesmodus_rotundus            GTCAGAGGTGCTGAAGATGGAACTGGCATCCAGGCCTCAGAGAACTGCTT                             ***   ** *  ** *****  * *** ******* ** **** ******Epomops_beuttikoferi         GTTTCGTTTCTTCAAAAACTCCTATTCTCCACTTCTGCTTAAGGACTGGAEidolon_helvum               ATTTCGTTTCTTCAAAAACTCCTATTCTCCACTTCTGCTTAAGGACTGGAHypsignathus_monstrosus      GTTTCGTTTCTTCAAAAACTCCTATTCTCCCCTTCTGCTTAAGGACTGGARousettus_aegyptiacus        GTTTCGTTTCTTCAAAAACTCCTATTCTCCACTTCTGCTTAAGGACTGGAMyotis_lucifugus             GTTCCGCTTCTTCCGAGACTCCTACTCCCCACTGCTGCTCAAGGCCTGGAMyotis_brandtii              GTTCCGCTTCTTCCGAGACTCCTACTCCCCACTGCTGCTCAAGGCCTGGAPteropus_alecto              GTTTCGTTTCTTCAAAAACTCCTATTCTCCACTTCTGCTTAAGGACTGGAArtibeus_jamaicensis         GTTTCGCTTCTTCAGGAACACTTATTCTCCGTTTCTGCTTAAGGACTGGACynopterus_sphinx            GTTTCGTTTCTTCAAAAACTCCTATTCTCCACTTCTGCTTAAGGACTGGADesmodus_rotundus            GTTTCACTTCTTCAGAGACGCCTATTCTCCGCTTCTGCTTAAGGACTGGA                              ** *  ******    ** * ** ** **  * ***** **** *****Epomops_beuttikoferi         TGCGACCAATTGTGATAGCAGTATTCGTGGGTGTTCTGTCATTCAGTACTEidolon_helvum               TGCGACCAATTGTGATAGCAGTATTCGTGGGTGTTCTGTCATTCAGTATTHypsignathus_monstrosus      TGCGACCAATTGTGATAGCAGTATTCGTGGGTGTTCTGTCATTCAGTACTRousettus_aegyptiacus        TGCGACCAATCGTGATAGCAGTATTCGTGGGTGTTCTGTCATTCAGTATTMyotis_lucifugus             TGCGGCCGATCGTGATGGCCGTGTTCGTGGGCGTTCTCTCCTTCAGCATCMyotis_brandtii              TGCGGCCGATCGTGATGGCCGTGTTCGTGGGCGTTCTCTCCTTCAGCATCPteropus_alecto              TGCGACCAATTGTGATAGCAGTATTCATGGGTGTTCTGTCATTCAGTATTArtibeus_jamaicensis         TGCGGCCGATCGTGGTAGCAGTATTTGTGGGGGTTCTCTCCTTCAGTGCCCynopterus_sphinx            TGCGGCCACTTGTGATAGCAGTATTCGTGGGTGTTCTGTCGTTCAGTATCDesmodus_rotundus            TGCGGCCGATCGTGATAGCAGTATTTGTGGGTGTCCTCTCATTCAGCATC                             **** **  * *** * ** ** **  **** ** ** ** *****    Epomops_beuttikoferi         GCAGTCCTGAACAAAGTAGAAATTGGATTGGATCAATCTCTTTCAGTGCCEidolon_helvum               GCAGTCCTGAACAAAGTAGAAATTGGATTGGATCAATCTCTTTCAGTGCCHypsignathus_monstrosus      GCAGTCCTGAACAAAGTAGAAATTGGATTGGATCAATCTCTTTCAGTGCCRousettus_aegyptiacus        GCAGTCCTGAACAAAGTAGAAATTGGATTGGATCAATCTCTTTCAGTGCCMyotis_lucifugus             GCCGTCCTGAACAAGGTGGAAATTGGATTGGACCAGTCTCTCTCAATGCCMyotis_brandtii              GCCGTCCTGAACAAGGTGGAAATTGGATTGGACCAGTCTCTCTCAATGCCPteropus_alecto              GCAGTCCTGAACAAAGTAGAAATTGGATTGGATCAATCTCTTTCAGTGCCArtibeus_jamaicensis         GCTGTCCTGAACAAAGTGGAAATTGGATTGGACCAGTCTCTTTCGATGCCCynopterus_sphinx            GCAGTCCTGAACAAAGTAGAAATTGGATTGGATCAATCTCTTTCAGTGCCDesmodus_rotundus            GCTGTCCTGAACAAAGTGGAGATTGGATTGGATCAGTCTCTTTCGATGCC                             ** *********** ** ** *********** ** ***** **  ****Epomops_beuttikoferi         AGATGACTCCTACGTGATGGATTATTTCAAGTCCCTCAGTCAGTACCTGCEidolon_helvum               AGATGACTCCTATGTGATGGATTATTTCAAGTCCCTCAGTCAGTACCTGCHypsignathus_monstrosus      AGATGACTCCTACGTGATGGATTATTTCAAGTCCCTCAGTCAGTACCTGCRousettus_aegyptiacus        AGATGACTCCTACGTGATGGATTATTTCAAGTCCCTCAGTCAGTACCTGCMyotis_lucifugus             AGAAGACTCCTACGTGATGGACTATTTCAGGTCCCTCAGCCGGTTCCTGCMyotis_brandtii              AGAAGACTCCTACGTGATGGACTATTTCAGGTCCCTCAGCCAGTTCCTGCPteropus_alecto              AGATGACTCCTACGTGATGGATTATTTCAAGTCCCTCAGTCAGTACCTGCArtibeus_jamaicensis         AGAAGACTCCTACGTGATGGATTATTTCAAGTCCCTCAGTCAGTACCTGCCynopterus_sphinx            AGATGACTCCTACGTGATGGATTATTTCAAGTCCCTCAGTCAGTACCTGCDesmodus_rotundus            AAAAGACTCCTACGTGATGGATTATTTCAAGGCCCTCAGTCAGTACCTGC                             * * ******** ******** ******* * ******* * ** *****Epomops_beuttikoferi         ATGCAGGCCCGCCCGTCTACTTTGTCCTGGAGGAAGGGCACAACTATACTEidolon_helvum               ACGCAGGCCCACCCGTCTACTTTGTCCTGAAGGAAGGACACAACTATACTHypsignathus_monstrosus      ATGCAGGCCCGCCCGTCTACTTTGTCCTGGAGGAAGGGCACAACTATACTRousettus_aegyptiacus        ATGCAGGCCCGCCCGTCTACTTCGTCCTGGAGGAAGGGCACAACTATACTMyotis_lucifugus             ACGCGGGCCCACCCGTCTACTTCGTGGTGGAGGAGGGGCTGGACTACACCMyotis_brandtii              ACGCGGGCCCACCCGTCTACTTCGTGGTGGAGGAGGGGCTGGACTACACAPteropus_alecto              ATGCAGGCCCACCCGTCTACTTTGTCCTGGAGGAAGGGCACAACTATACTArtibeus_jamaicensis         ACGCGGGCCCGCCTGTCTACTTTGTCCTGGAGGAAGGGCACGACTACACTCynopterus_sphinx            ATGCAGGCCCGCCTGTCTACTTTGTCCTGGAGGAAGGGCACAACTATACTDesmodus_rotundus            ACGCGGGCCCGCCTGTCTACTTCGTCCTGGAGGAGGGGCATGACTACACA                             * ** ***** ** ******** **  ** **** ** *   **** ** Epomops_beuttikoferi         TCTCTGGCTGGGCAGAACATAGTATGCGGGGGCGTGGGCTGCAACAACGAEidolon_helvum               TCTCTGGCAGGACAGAACATAGTGTGCGGGGGCATGGGCTGCAACAACGAHypsignathus_monstrosus      TCTCTGGCTGGGCAGAACATAGTATGCGGGGGCGTGGGCTGCAACAACGARousettus_aegyptiacus        TCTCTGGCAGGGCAGAACATAGTGTGCGGGGGCATGGGCTGCAACAACGAMyotis_lucifugus             TCCCTGCAGGGGCAGAACCTGGTGTGCGGGGGCATGGGCTGCAACAACGAMyotis_brandtii              TCCCTGCAGGGGCAGAACCTGGTGTGCGGGGGCATGGGCTGCAACAACGAPteropus_alecto              TCTCTGGCAGGGCAGAACATGGTGTGCGGGGGCGTGGGCTGCAACAGCGAArtibeus_jamaicensis         TCTCTGAAGGGGCAGAACATGGTGTGCGGGGGCATGGGCTGCAACAACGACynopterus_sphinx            TCTCTGGCAGGGCAGAACATAGTGTGTGGAGGCGTGGGCTGCAACAACGADesmodus_rotundus            TCTCTGAAGGGGCAGAACATGGTGTGCGGGGGCGTGGGCTGCAACAACGA                             ** ***   ** ****** * ** ** ** *** ************ ***Epomops_beuttikoferi         CTCTCTGGTGCAGCAGATCTTCAACGCGGCGGAGCTGGACAACTATACCCEidolon_helvum               CTCCTTGGTGCAGCAGATCTTCAACGCAGCCGAGCTGGACAACTATACCCHypsignathus_monstrosus      CTCCCTGGTGCAGCAGATCTTCAACGCGGCGGAGCTGGACAACTATACCCRousettus_aegyptiacus        CTCCCTGGTGCAGCAGATCTTCAACGCGGCGGAGCTGGACAACTATACCCMyotis_lucifugus             CTCGCTGGTGCAGCAGCTGTTCGACGCGGCCGAGCTGGATGCCTACACCCMyotis_brandtii              CTCGCTGGTGCAGCAGCTGTTCGACGCGGCTGAGCTGGATGCCTACACCCPteropus_alecto              CTCCCTGGTGCAGCAGATCTTCAACGCGGCCGAGCTGGAAAACTATACCCArtibeus_jamaicensis         CTCGCTGGTGCAGCAGATATACGCAGCGGCCGAGCTGGACAACTATACCCCynopterus_sphinx            CTCCCTGGTGCAGCAGATCTTCAACGCGGCCCAGCTGGACAACTATACCCDesmodus_rotundus            CTCGCTGGTGCAGCAGATTTACGAAGCGGCTGAGCTGGACAACTATACCC                             ***  *********** * * *   ** **  *******   *** ****Epomops_beuttikoferi         GGATAGGCTTTGCTCCCTCATCCTGGATTGACGATTACTTTGATTGGATTEidolon_helvum               GGATAGGTTTTGCTCCCTCTTCCTGGATTGATGATTACTTTGATTGGATTHypsignathus_monstrosus      GGATAGGCTTTGCTCCCTCATCCTGGATTGACGATTACTTTGATTGGATTRousettus_aegyptiacus        GAATAGGCTTTGCTCCCTCCTCCTGGATTGACGATTACTTTGATTGGATTMyotis_lucifugus             GGATCGGCTTTGCCCCCTCGTCCTGGATCGACGACTACTTCGACTGGGTCMyotis_brandtii              GGATCGGCTTCGCCCCCTCGTCCTGGATCGACGACTACTTCGACTGGGTCPteropus_alecto              GGATAGGCTTTGCTCCCTCATCCTGGATTGACGATTACTTTGATTGGATTArtibeus_jamaicensis         GAATAGGCTTTGCGCCCTCGTCCTGGATTGATGATTACTTCGACTGGGTTCynopterus_sphinx            GGATAGGCTTTGCTCCCTCATCCTGGATTGATGATTACTTTGATTGGATTDesmodus_rotundus            GAATAGGCTTTGCTCCCTCGTCCTGGATTGACGATTACTTTGATTGGATT                             * ** ** ** ** ***** ******** ** ** ***** ** *** * Epomops_beuttikoferi         AAGCCACAGTCTTCTTGCTGTAGAGTCTACAATAGCACTGACCAGTTCTGEidolon_helvum               AAGCCACAATCTTCTTGCTGTAGAGTCTACAATAACACTGACCAATTCTGHypsignathus_monstrosus      AAGCCACAGTCTTCTTGCTGTAGAGTCTACAATAGCACTGACCAGTTCTGRousettus_aegyptiacus        AAGCCACAGTCTTCTTGCTGTAGAGTCTACAATGGCACTGACCGGTTCTGMyotis_lucifugus             AAGCCGCAGTCCTCGTGCTGCCGGGTCCACAACGGCTCCGGGCAGTTCTGMyotis_brandtii              AAGCCGCAGTCCTCGTGCTGCCGGGTCCACAGCAGCTCCGGGCAGTTCTGPteropus_alecto              AAGCCACAGTCTTCTTGCTGTAGAGTCTACAATGGCACTGACCAGTTCTGArtibeus_jamaicensis         AAGCCGCAGTCTTCTTGCTGTAGAGTCTACAATGGCACAGATCGGTTCTGCynopterus_sphinx            AAGCCGCAGTCTTCTTGCTGTAGAGTCTACAATAGCACTGACCAGTTCTGDesmodus_rotundus            AAGCCGCAGTCTTCTTGCTGTAGAGTCTACAATAGCACTGATCGGTTCTG                             ***** ** ** ** *****  * *** ***    * * *  *  *****Epomops_beuttikoferi         CAATGCTTCAGTGGCTGACCCTGCCTGCATCCGCTGCAGGCCTCTGACTCEidolon_helvum               CAACGCTTCAGTGGTTGACCCTGCCTGCATCCGCTGCAGGCCTCTGACTCHypsignathus_monstrosus      CAATGCTTCAGTGGCTGACCCTGCCTGCATCCGCTGCAGGCCTCTGACTCRousettus_aegyptiacus        CAATGCTTCAGTGGCCGACCCTGCCTGCATCCGCTGCAGGCCTCTGACTCMyotis_lucifugus             CAACGCCTCGGTGGCCGACCCTGGCTGCATCCGCTGCAGGCCTCTGACTCMyotis_brandtii              CAACGCCTCGGTGGCCGACCCTGGCTGCATCCGCTGCAGGCCTCTGACTCPteropus_alecto              CAACGCTTCAGTGGCTGACCCTACCTGCATCCGCTGCAGGCCTCTGACTCArtibeus_jamaicensis         CAATGCTTCAGTGGCTGACCCTGCCTGCGTCCGATGTCGGCCCCTGACCCCynopterus_sphinx            CAACGCTTCAGTGGCTGACCCTGCCTGCATCCGCTGCAGGCCTCTGACCCDesmodus_rotundus            CAATGCTTCAGTGGCTGACCCTGCCTGCGTCCGATGCCGGCCCCTGACTC                             *** ** ** ****  ******  **** **** **  **** ***** *Epomops_beuttikoferi         CCGAGGGCAAACAGAGGCCTCAGGGTGGAGACTTCATGAAATTCCTGCCCEidolon_helvum               CTGAGGGCAAACAGAGGCCTCAGGGTGGAGACTTCATGAGATTCCTGCCTHypsignathus_monstrosus      CCGAGGGCAAACAGAGGCCTCAGGGTGGAGACTTCATGAAATTCCTGCCCRousettus_aegyptiacus        CCGAGGGCAAGCAGAGGCCTCAGGGTAGAGACTTCATGAGATTCCTGCCCMyotis_lucifugus             CCGAGGGCAAACAGCGGCCCCAGGGGGGAGACTTCATGCGGTTCCTGCCCMyotis_brandtii              CCGAGGGCAAACAGCGGCCCCAGGGGGGAGACTTCATGCGGTTCCTGCCCPteropus_alecto              CCGAGGGCAAACAGAGGCCTCAGGGTGGAGACTTCATGAGATTCCTGCCCArtibeus_jamaicensis         CAGAGGGCAAACAGAGGCCTCAGGGTGGAGACTTCATGAGATTCCTGCCCCynopterus_sphinx            CCGAGGGAAAACAGAGGCCTCAGGGTGGAGACTTCATGAGATTCCTGCCCDesmodus_rotundus            CAGAGGGCAAACAGAGGCCTCAGGGTGGAGACTTCATGAGATTCCTGCCC                             * ***** ** *** **** *****  ***********   ******** Epomops_beuttikoferi         ATGTTCCTTTCCGATAACCCGAACCCCAAGTGTGGCAAAGGGGGACATGCEidolon_helvum               ATGTTCCTTTCCGATAACCCGAACCCCAAGTGTGGCAAAGGGGGACATGCHypsignathus_monstrosus      ATGTTCCTTTCCGATAACCCGAACCCCAAGTGTGGCAAAGGGGGACATGCRousettus_aegyptiacus        ATGTTCCTTTCCGATAACCCGAACCCCAAGTGTGGCAAAGGGGGACATGCMyotis_lucifugus             ATGTTCCTCTCCGACAACCCGAACCCCAAGTGTGGCAAAGGGGGACATGCMyotis_brandtii              ATGTTCCTCTCCGACAACCCGAACCCCAAGTGTGGCAAAGGGGGACATGCPteropus_alecto              ATGTTCCTTTCTGATAACCCGAATCCCAAGTGTGGCAAAGGGGGACATGCArtibeus_jamaicensis         ATGTTCCTTTCTGATAACCCGAACCCCAAGTGCGGCAAAGGGGGACATGCCynopterus_sphinx            ATGTTCCTTTCTGATAACCCGAACCCCAAGTGTGGCAAAGGGGGACATGCDesmodus_rotundus            ATGTTCCTTTCTGATAATCCGAACCCCAAGTGTGGCAAAGGGGGACATGC                             ******** ** ** ** ***** ******** *****************Epomops_beuttikoferi         TGCTTATGGGTCAGCAGTTAACATCCTTGGTAATGACACGGGTGTCGGAGEidolon_helvum               TGCTTATGGATCAGCAGTTAACATCCTTGGTAATGACACGGGTGTTGGAGHypsignathus_monstrosus      TGCTTATGGGTCAGCAGTTAACATCCTTGGTAATGACACGGGTGTCGGAGRousettus_aegyptiacus        TGCTTATGGGTCAGCAGTTAACATCCTTGGTAATGACACGGGTGTCGGAGMyotis_lucifugus             TGCCTACGGGTCGGCCGTTAACATCCTGGGCAATGACACAGGTGTGGGCGMyotis_brandtii              TGCCTACGGGTCGGCCGTTAACATCCTGGGCAATGACACAGGTGTGGGCGPteropus_alecto              TGCTTATGGGTCAGCAGTTAACATCCTTGGTAATGACACGAGTGTCGGAGArtibeus_jamaicensis         CGCTTATGGGTCGGCAGTAAACATCCTTGACAACGACACAGGTGTTGGAGCynopterus_sphinx            TGCTTATGGGTCAGCAGTTAACATCCTTGGTAATGACACGGGCGTCGGAGDesmodus_rotundus            TGCTTACGGGTCGGCAGTTAACATCCTTGACGACAACACGGGTGTCGGAG                              ** ** ** ** ** ** ******** *   *  ****  * ** ** *Epomops_beuttikoferi         CCACATACTTCATGACCTACCACACCGTGCTTCAGACCTCTGCTGACTTTEidolon_helvum               CCACATACTTCATGACCTACCACACCGTGCTTCAGACCTCTGCTGACTTTHypsignathus_monstrosus      CCACATACTTCATGACCTACCACACCGTGCTTCAGACCTCTGCTGACTTTRousettus_aegyptiacus        CCACGTACTTCATGACCTACCACACCGTGCTTCAGACCTCTGCTGACTTTMyotis_lucifugus             CCACCTACTTCATGACCTACCACACCGTGCTCCAGACCTCTGCTGACTTCMyotis_brandtii              CCACCTACTTCATGACCTACCACACCGTGCTCCAGACCTCTGCTGACTTCPteropus_alecto              CCACGTACTTCATGACCTACCACACCGTGCTTCAGACCTCTGCTGACTTTArtibeus_jamaicensis         CCACGTACTTCATGACGTACCACACCGTGCTTCAGACTTCTGCCGACTTCCynopterus_sphinx            CCACGTACTTCATGACCTACCACACCGTGCTTCAGACCTCTGCTGACTTTDesmodus_rotundus            CCACATACTTCATGACCTACCACACCGTGCTGCAGACCTCTGCGGACTTC                             **** *********** ************** ***** ***** ***** Epomops_beuttikoferi         ATTGATGCCATGAGAAAAGCTCGCCTCATCGCTGATAATATCACCAAAACEidolon_helvum               ATTGATGCCATGAGAAAAGCTCGCCTCATCGCTGGTAACATCACCAAAACHypsignathus_monstrosus      ATTGATGCCATGAGAAAAGCTCGCCTCATCGCTGATAATATCACCAAAACRousettus_aegyptiacus        ATTGATGCCATGAGAAAAGCTCGCCTCATCGCTGATAATATCACCAAAACMyotis_lucifugus             ATCGACGCCATGAGAAAGGCCCGGCTCATTGCTGGTAACATCACCCAGACMyotis_brandtii              ATCGACGCCATGAGAAAGGCCCGGCTCATTGCTGGTAACATCACCCAGACPteropus_alecto              ATTGACGCCATGAGAAAAGCTCGCCTCATCGCTGCTAACATCACCAAAACArtibeus_jamaicensis         ATTGATGCCATGAAAAAAGCCCGCCTTATTGCTGGTAACATCACCAAAACCynopterus_sphinx            ATTGACGCCATGAGAAAAGCTCGCCTCATCGCTGGTAACATCACCAAAACDesmodus_rotundus            ATTGATGCCATGGAAAAAGCCCGCCTCGTTGCTGGTAACATCACCAAAAC                             ** ** ******  *** ** ** **  * **** *** ****** * **Epomops_beuttikoferi         CATGAGCCAGGAAGGAAGTAACCACCGTGTATTCCCATACAGTGTGTTCTEidolon_helvum               CATGAGCCAGGAAGGAAGTAACCACCATGTATTCCCATACAGTGTGTTCTHypsignathus_monstrosus      CATGAGCCAGGAAGGAAGTAACCACCGTGTATTCCCATACAGTGTGTTCTRousettus_aegyptiacus        CATGAGCCAGGAAGGAAGTAACCACCGTGTATTCCCATACAGCGTGTTTTMyotis_lucifugus             CATGAGCCAGGGAGGAAGCAACTACCGCGTGTTTCCATACAGTGTGTTCTMyotis_brandtii              CATGAGCCAGGGAGGAAGCAACTACCGCGTATTTCCATACAGTGTGTTCTPteropus_alecto              CATGAGCCAGGAAGGAAGTAACCACCATGTATTCCCATACAGTGTGTTCTArtibeus_jamaicensis         CATGAGCCAGGGAGGAAGTAATTACCGCGTGTTCCCGTACAGTGTGTTCTCynopterus_sphinx            CATGAGCCAGGAAGGAAGTAACCACAATGTATTCGCATACAGTGTGTTCTDesmodus_rotundus            CATGAGCCAGGGAGGAAGTCGTTACCGCGTGTTCCCATACAGTGTGTTCT                             *********** ******     **   ** **  * ***** ***** *Epomops_beuttikoferi         ATGTCTTTTACGAACAGTACCTGACTATTATTGATGACACGATCTTTAACEidolon_helvum               ATGTCTTTTACGAACAGTACCTGACCATTATTGATGACACGATCTTTAACHypsignathus_monstrosus      ATGTCTTTTACGAACAGTACCTGACTATTATTGATGACACGATCTTTAACRousettus_aegyptiacus        ATGTCTTTTACGAACAGTACCTGACTATTATTGATGACACGATCTTTAACMyotis_lucifugus             ACGTCTTCTACGAGCAGTACCTGACCATCATAGAAGACACCGTCTTCAACMyotis_brandtii              ACGTCTTCTACGAGCAGTACCTGACCATCATAGAGGACACCGTCTTCAACPteropus_alecto              ATGTCTTTTACGAACAGTACCTGACTATTATCGATGACACGATCTTTAACArtibeus_jamaicensis         ATGTCTTCTACGAGCAGTACCTGACCATTATTGACGACACAATCTTCAACCynopterus_sphinx            ATGTCTTTTACGAACAGTACCTAACTATTATTGATGACACGATCTTTAACDesmodus_rotundus            ATGTCTTCTACGAACAGTACCTGACCATTATTGACGACACGATCTTTAAC                             * ***** ***** ******** ** ** ** ** *****  **** ***Epomops_beuttikoferi         CTCTGCGTGTCCCTTGGAGCGATCTTTTTGGTGACCGTAGTTCTGCTGGGEidolon_helvum               CTCTGCGTGTCCCTGGGAGCAATCTTTTTGGTGACCGTGGTTCTCCTGGGHypsignathus_monstrosus      CTCTGCGTGTCCCTTGGAGCGATCTTTTTGGTGACCGTAGTTCTGCTGGGRousettus_aegyptiacus        CTCTGCGTGTCCCTTGGAGCGATCTTTTTGGTGACCGTGGTTCTGCTGGGMyotis_lucifugus             CTCTGCGTGTCCCTGGGGGCCATCTTCCTGGTGACCGTGGTGCTGCTGGGMyotis_brandtii              CTCTGCGTGTCCCTGGGGGCCATCTTCCTGGTGACCGTGGTGCTGCTGGGPteropus_alecto              CTCTGCGTGTCCCTGGGAGCGATCTTTCTGGTGACTGTGGTTCTCCTGGGArtibeus_jamaicensis         CTTTGCGTCTCCCTGGGAGCCATCTTTTTGGTGACCGTGGTTCTCCTGGGCynopterus_sphinx            CTCTGCGTGTCCCTGGGAGCAATCTTTTTGGTGACCGTGGTTCTCCTGGGDesmodus_rotundus            CTTGGCGTGTCCCTGGGGGCCATCTTCGTGGTGACCGTGGTTCTCCTGGG                             **  **** ***** ** ** *****  ******* ** ** ** *****Epomops_beuttikoferi         CTGTGAGCTGTGGTCTGCAGTGATCATGTGTGTCACCATCGCCATGATCTEidolon_helvum               CTGTGAGCTGTGGTCTGCAGTGATCATGTGTGTCACCATCGCCATGATCTHypsignathus_monstrosus      CTGTGAGCTGTGGTCTGCAGTGATCATGTGTGTCACCATCGCCATGATCTRousettus_aegyptiacus        CTGTGAGCTGTGGTCTGCAGTGATCATGTGTGTCACCATCGCCATGATCTMyotis_lucifugus             CTGCGAGCTGTGGTCTGCGGTGATCATGTGCGTCACCATCGCCATGATCCMyotis_brandtii              CTGCGAGCTGTGGTCTGCGGTGATCATGTGCGTCACCATCGCCATGATCCPteropus_alecto              CTGTGAACTGTGGTCTGCAGTGATCATGTGTGTCACCATCGCCATGATCTArtibeus_jamaicensis         CTGTGAACTGTGGTCTGCAGTGATCATGTGTGTCACCATCGCCATGATCCCynopterus_sphinx            CTGTGAGCTGTGGTCTGCAGTGATCATGTGTGTCACCATCGCCATGATCTDesmodus_rotundus            CTGTGAACTGTGGTCTGCGGTGATCATGTGTGTCACCATCGCCATGATCC                             *** ** *********** *********** ****************** Epomops_beuttikoferi         TAGTCAACATGTTTGGTGTCATGTGGCTGTGGGGCATCAGTCTGAACGCAEidolon_helvum               TAGTCAACATGTTTGGCGTCATGTGGCTGTGGGGCATCAGTCTGAACGCAHypsignathus_monstrosus      TAGTCAACATGTTTGGTGTCATGTGGCTGTGGGGCATCAGTCTGAACGCARousettus_aegyptiacus        TAGTCAACATGTTTGGTGTCATGTGGCTGTGGGGCATCAGTCTGAACGCAMyotis_lucifugus             TGGTCAACATGTTTGGTGTCATGTGGCTGTGGGGCATCAGCCTGAACGCAMyotis_brandtii              TGGTCAACATGTTTGGTGTCATGTGGCTGTGGGGCATCAGCCTGAACGCGPteropus_alecto              TAGTCGACATGTTTGGCATCATGTGGCTGTGGGGCATCAGTCTGAATGCAArtibeus_jamaicensis         TGGTCAACATGTTTGGCGTCATGTGGCTGTGGGGCATCAGCCTGAACGCACynopterus_sphinx            TAGTCAACATGTTTGGCGTCATGTGGCTGTGGGGCATCAGTCTGAACGCADesmodus_rotundus            TGGTCAACATGTTCGGCGTCATGTGGCTGTGGGGCATCAGCCTGAACGCG                             * *** ******* **  ********************** ***** ** Epomops_beuttikoferi         GTTTCCTTGGTCAACTTGGTTATGAGCTGTGGCATCTCCGTGGAGTTCTGEidolon_helvum               GTTTCCTTGGTCAACTTGGTTATGAGCTGTGGCATTTCCGTGGAGTTCTGHypsignathus_monstrosus      GTTTCCTTGGTCAACTTGGTTATGAGCTGTGGCATCTCCGTGGAGTTCTGRousettus_aegyptiacus        GTTTCCTTGGTCAACTTGGTTATGAGCTGTGGCATCTCCGTGGAGTTCTGMyotis_lucifugus             GTCTCCCTGGTCAACCTGGTCATGAGCTGCGGCATCTCGGTGGAGTTCTGMyotis_brandtii              GTCTCCCTGGTCAACCTGGTCATGAGCTGCGGCATCTCGGTGGAGTTCTGPteropus_alecto              GTTTCCTTGGTCAACTTGGTTATGAGCTGTGGCATTTCCGTGGAGTTCTGArtibeus_jamaicensis         GTTTCCCTGGTCAACTTGGTCATGAGCTGTGGCATCTCCGTGGAGTTCTGCynopterus_sphinx            GTTTCCTTGGTCAACTTGGTTATGAGCTGTGGCATTTCCGTGGAGTTCTGDesmodus_rotundus            GTCTCCCTGGTCAACTTGGTCATGAGTTGTGGCATCTCTGTGGAGTTCTG                             ** *** ******** **** ***** ** ***** ** ***********Epomops_beuttikoferi         CAGCCACATAACGAGAGCATTCACAGTGAGCGCGAAGGGAAGCCGTGTGGEidolon_helvum               CAGCCACATAACGAGAGCATTCACAGTGAGTGCAAAGGGAACCCGTGTGGHypsignathus_monstrosus      CAGCCACATAACGAGAGCATTCACAGTGAGCGCGAAGGGAAGCCGTGTGGRousettus_aegyptiacus        CAGCCACATAACGAGAGCATTCACAGTGAGTGCAAAGGGAAGCCGTGTGGMyotis_lucifugus             CTCCCACATCACCAGGGCCTTCACAGTGAGCGCGAAGGGCAGCCGCGTGGMyotis_brandtii              CTCCCACATCACCAGGGCCTTCACAGTGAGCGCGAAGGGCAGCCGCGTGGPteropus_alecto              CAGCCACATAACCAGAGCATTCACAGTGAGTGCGAAGGGAAGCCGTGTGGArtibeus_jamaicensis         CAGCCACGTAACGAGAGCGTTCACCATGAGTGCAAAGGGAAGCCGCGTGGCynopterus_sphinx            CAGCCACATAACGAGAGCATTCACAGTGAGTGCAAAGGGAAGTCGTGTGGDesmodus_rotundus            CAGCCACATAACGAGAGCGTTCACCGTGAGTGCAAAGGGAAGCCGTGTGG                             *  **** * ** ** ** *****  **** ** ***** *  ** ****Epomops_beuttikoferi         AACGGGCAGAAGAGGCGCTCTCTCACATGGGCAGTTCTGTATTCAGTGGAEidolon_helvum               AACGGGCAGAAGAGGCGCTCTCTCACATGGGCAGTTCTGTATTCAGTGGAHypsignathus_monstrosus      AACGGGCAGAAGAGGCGCTCTCTCACATGGGCAGTTCTGTATTCAGTGGARousettus_aegyptiacus        AACGGGCAGAAGAGGCGCTCTCTCACATGGGCAGTTCTGTATTCAGTGGAMyotis_lucifugus             CGCGTGCAGAGGAGGCGCTCTCCCACATGGGCAGCTCCGTATTCAGTGGAMyotis_brandtii              CGCGTGCAGAGGAGGCGCTCTCCCACATGGGCAGCTCCGTATTCAGTGGAPteropus_alecto              AACGGGCAGAAGAGGCACTCTCTCACATGGGCAGTTCTGTATTCAGTGGAArtibeus_jamaicensis         AACGTGCGGAAGAGGCGCTCTCCCACATGGGCAGTGCTGTATTCAGTGGACynopterus_sphinx            AACGGGCAGAAGAGGCGCTCTCTCACATGGGCAGTTCTGTATTCAGTGGADesmodus_rotundus            AACGTGCGCAAGAGGCGCTCTCCCACATGGGCAGCTCTGTATTCAGTGGA                               ** **  * ***** ***** ***********  * ************Epomops_beuttikoferi         ATCACACTTACAAAGTTTGGAGGGATTGTGGTATTGGCCTTTGCCAAATCEidolon_helvum               ATCACACTTACAAAGTTTGGAGGGATTGTGGTATTGGCCTTTGCCAAATCHypsignathus_monstrosus      ATCACACTTACAAAGTTTGGAGGGATTGTGGTATTGGCCTTTGCCAAATCRousettus_aegyptiacus        ATCACACTTACAAAGTTTGGAGGGATTGTGGTATTGGCCTTTGCCAAATCMyotis_lucifugus             ATCACACTTACAAAATTTGGAGGGATTGTGGTGTTGGCCTTTGCCAAGTCMyotis_brandtii              ATCACACTTACAAAATTTGGAGGGATTGTGGTGTTGGCCTTTGCCAAGTCPteropus_alecto              ATCACACTTACAAAGTTTGGAGGGATTGTGGTATTGGCCTTTGCCAAATCArtibeus_jamaicensis         ATCACACTTACTAAATTTGGAGGGATTGTGGTGTTGGCCTTTGCCAAGTCCynopterus_sphinx            ATCACACTTACAAAGTTTGGAGGGATTGTGGTATTGGCCTTTGCCAAATCDesmodus_rotundus            ATCACACTTACAAAATTTGGAGGGATTGTGGTGTTGGCCTTTGCCAAATC                             *********** ** ***************** ************** **Epomops_beuttikoferi         TCAAATTTTCCAGATATTTTACTTCAGGATGTATTTAGCTATGGTCTTACEidolon_helvum               TCAAATTTTCCAGATATTTTACTTCAGGATGTATTTAGCTATGGTCTTATHypsignathus_monstrosus      TCAAATTTTCCAGATATTTTACTTCAGGATGTATTTAGCTATGGTCTTACRousettus_aegyptiacus        TCAAATTTTCCAGATATTTTACTTCAGGATGTATTTAGCTATGGTCTTACMyotis_lucifugus             TCAGATCTTCCAGATATTTTACTTCAGGATGTACCTAGCTATGGTCTTGCMyotis_brandtii              TCAGATCTTCCAGATATTTTACTTCAGGATGTACCTAGCTATGGTCTTGCPteropus_alecto              TCAAATTTTCCAGATATTTTACTTCAGGATGTATTTAGCTATGGTCTTGCArtibeus_jamaicensis         TCAAATTTTCCAGATATTTTACTTCAGGATGTATTTAGCTATGGTCTTACCynopterus_sphinx            TCAAATTTTCCAGATATTCTACTTCAGGATGTATTTAGCTATGGTCTTACDesmodus_rotundus            TAAAATTTTCCAGATATTTTACTTCAGGATGTATTTAGCTATGGTCTTAC                             * * ** *********** **************  *************  Epomops_beuttikoferi         TGGGAGCCAGTCATGGACTAATATTCCTTCCTGTCTTACTCAGTTATATAEidolon_helvum               TGGGAGCCAGTCATGGATTAATATTCCTCCCTGTCTTACTCAGTTATATAHypsignathus_monstrosus      TGGGAGCCAGTCATGGACTAATATTCCTTCCTGTCTTACTCAGTTATATARousettus_aegyptiacus        TGGGAGCCAGTCATGGACTAATATTCCTCCCTGTCTTACTCAGTTATATAMyotis_lucifugus             TGGGAGCCGCGCACGGGCTGGTCTTCCTTCCTGTCCTCCTCAGCTACATAMyotis_brandtii              TGGGAGCCGCGCACGGGCTGGTCTTCCTTCCTGTCCTCCTCAGCTACATAPteropus_alecto              TGGGAGCCAGTCATGGATTAATATTCCTCCCTGTCTTACTCAGTTATATAArtibeus_jamaicensis         TGGGAGCCACTCATGGACTGATATTCCTTCCTGTCTTACTCAGTTACATACynopterus_sphinx            TGGGAGCCAGTCATGGATTAATATTCCTCCCTGTCTTACTCAGTTATATADesmodus_rotundus            TGGGAGCCACTCATGGATTGATATTCCTTCCTGTCTTACTTAGTTATATA                             ********   ** **  *  * ***** ****** * ** ** ** ***Epomops_beuttikoferi         GGACCATCAATAAATAAAGCCAAAAGTTTGGCTACTCAAGAGCGGTATAAEidolon_helvum               GGACCATCAATAAATAAAGCCAAAAGTTTGGCTACTCAAGAGCGATATAAHypsignathus_monstrosus      GGACCATCAATAAATAAAGCCAAAAGTTTGGCTACTCAAGAGCGGTATAARousettus_aegyptiacus        GGACCATCAATAAATAAAGCCAAAAGTTTGGCTACTCAAGAGCGATATAAMyotis_lucifugus             GGACCATCCGTAAATAAAGCCAAAAGCCTGGCCACGCAACAGCGACACAGMyotis_brandtii              GGACCATCCGTAAATAAAGCCAAAAGCCTGGCCACGCAACAGCGACACAGPteropus_alecto              GGACCATCAATAAATAAAGCCAAAAGTTTGGCTACTCAAGAGCGATATCAArtibeus_jamaicensis         GGACCATCAATAAATAAAGCCAAAAATTTGGCCGCTCGAGAGCGATATAACynopterus_sphinx            GGACCATCAATAAATAAAGCCAAAAGTTTAGCTACTCAAGAGCGATATAADesmodus_rotundus            GGGCCATCGATAAATAAAGCCAAAAGTTTGGCCGCTCGAGAGCGATACAG                             ** *****  ***************   * **  * * * ****  *   Epomops_beuttikoferi         AGGTACAGAGAGAGAACAACTCCTAAATTTCTAAEidolon_helvum               AGGTACAGAGAGAGAACAACTCCTAAATTTCTAAHypsignathus_monstrosus      AGGTACAGAGAGAGAACAACTCCTAAATTTCTAARousettus_aegyptiacus        AGGTACAGAGAGAGAACAACTCCTAAATTTCTAAMyotis_lucifugus             AGGCACAGAGAGAGAGCAGCTC---AATTCTTAGMyotis_brandtii              AGGCACAGAGAGAGAGCAGCTC---AATTTCTAGPteropus_alecto              AGGTACAGAGAGAGAACGACTCCTCAATTTCTAAArtibeus_jamaicensis         AGGCACAGAGAGAGAACAACTCCTCAATTTCTAACynopterus_sphinx            AGGTACAGAGAGAGAACAACTCCTAAATTTCTAADesmodus_rotundus            AGGCACAGAGAGAGAACAACTCCTAAATTTCTAA                             *** *********** *  ***   ****  ** 
