# Supplementary material for: Filovirus receptor NPC1 contributes to species-specific patterns of ebolavirus susceptibility in bats
Source: eLife. 2015 Dec 23;4:e11785. doi: 10.7554/eLife.11785 (PMC4709267; doi:10.7554/eLife.11785)
Supplement: Supplementary file 5. — Multiple sequence alignment showing synteny between sequences flanking filovirus NP-derived EVEs in the indicated bat genomes. (A) The region flanking the 5’ end of the EVE ortholog. (B) Amino acid sequence alignment of a conserved region within exogenous filovirus NP and the putative EVE ORFs found of these sequences (note: the E. helvum sequence in deleted in this region and is not shown). (C) Region flanking the 3’ end of the EVE ortholog. Nucleotide insertions that were present in only one sequence are not shown. EVE sequence is highlighted in grey. Accession numbers for EVE sequences are as follows; M. brandtii (ANKR01230743.1); M. lucifugus (AAPE02014310.1); M. davidii (ALWT01026193.1); E. fuscus (ALEH01076399.1); E. helvum (AWHC01132512.1); P. vampyrus (ABRP02039678.1); P. alecto (ALWS01163349.1); P. parnellii (AWGZ01223755.1). DOI: http://dx.doi.org/10.7554/eLife.11785.021 [file elife-11785-supp5.zip › Supplementary file 5.rtf]

Supplementary file 5


a) 5' flanking site

Consensus     CCACATTTCTTCATCTGTAAAATAGCATAATATTAGTGTCTACTTCATGTGATTGTTGTG
M.davidii     TCTAATTTCTTCGTCTGTAAAATAGCA---TATTAGTGTCTACTTCACGTGATTGCTGTG
M.brandtii    TCTCATTTCTTCGTCTGTAAAATAGCA---TATTAGTGTCTACTTCAAGTGATTGCTGTG
M.lucifugus   TCGCATTTCTTCGTCTGTAAAATAGCA---TATTAGTGTCTACTTCACATGATTGCTGTG
E.fuscus      TCGCATTTCTTTGTTTGTAAAATAGCA---TATTAGTGTCTACTTCACGTGATTGTTGTG
E.helvum      CCACATTTCTTCATCTGTAAAATAGCATAATAATAGTGTCTACTTCATGTGATTGTTGTG
P.vampyrus    CCACATTTCTTCATCTGTAGAATAGCTTAATAATAGTGTCTACTTCATGTGATTGTTGTA
P.alecto      CCACATTTCTTCATCTGTAGAATAGCTTAATAATAGTGTCTACTTCATGTGATTGTTGTA
P.parnellii   CAGCATTTCTTCATCTGTAAAATTGCATAATATTAGTGTCTACTTCATCTGATCATCACG

Consensus     AGAATGGTAAGAGGTTAAGACAAATAAATAGCTTACCACAGTGCCTGGCTGCCATCATTG
M.davidii     AGAAGGGTAAGAGGTTAAGACAAATAAGTGGCTTACCACAGTGCCCGGCTGCCATCATTG
M.brandtii    AGAAGGGTAAGAGGTTAAGACAAATAAGTGGCTTACCACAGTGCCTGGCTGCCATCATTG
M.lucifugus   AGAAGGGTAAGAGGTTAAGACAAATAAGTGGCTTACCACAGTGCCCGGCTGCCATCATTG
E.fuscus      AGAATGGTAGGAGGTTAAGACAAATAAATGGCTTACCACAGTGCCTGGCTGCCATCATTG
E.helvum      AGAATGATAAGAAGT-AATACAAAGAAATAACTTACTACAGTGTCTGGCTGCTGCCATTT
P.vampyrus    AGAATGATAAGAAGT-AATACAAAGAAATAGCTTACTACAGTGCCTGGCTGCTGCCGTTT
P.alecto      AGAATGATAAGAAGT-AATACAAAGAAATAGCTTACTACAGTGCCTGGCTGCTGCCGTTT
P.parnellii   AGAGCGCTAAGAGGT-AATACAAATAAATAGCTTACCGCAGTGCTTGGCTGCCATTGTTT

Consensus     GGATTCCCCCAGAAGCAGACCCTGGGACAAGGATTCAAATACAAGTAGTTTAGGAAGAGC
M.davidii     GGATACTCCCAGAAGCAGA-CCTGGGACAAGGATTCAAATACAAGTAGTTTAGGAAGAGC
M.brandtii    GGATTCCCCCAGAAGCAGT-CCTGGGACAAGGATTCAAATACAAGTAGTTTAGGAAGAGC
M.lucifugus   GGTTACTCCCAGAAGCAGA-CCTGGGACAAGGATTCAAATACAAGTAGTTTAGGAAGAGC
E.fuscus      GGATTCCCCGAGAAGCAGACCCTGGGACAAGGGTTCAAATACAAGTAGTTTAGGAAGAGC
E.helvum      GGGTTTCCC-AGAAGCAGACCCTGGGACAAGGATTCAAGTGCAAGTAGCTTATTTAGGCG
P.vampyrus    GGGTTTCCC-AGAAGCAGACCCTGGGACAAGGATTCAAGTGCAAGTAGTTTATTTAGGAG
P.alecto      GGGTTTCCC-AGAAGCAGACCCTGGGACAAGGATTCAAGTGCAAGTAGTTTATTTAGGAG
P.parnellii   GGATTCCCCCAGAAGCAAACCCGGGGACAAGGATACAGGTGCAAGTAGCCTACACAAGGG

	                                        -> Start of Filovirus NP
Consensus     ATCAAGCTATTAAACACCTAGTTGTGGGGAACGATGCATTCCATTCTTAATGCCGAATTA
M.davidii     ATCAAGCTATT----ACCTAGTTGTG---AACGATGCATTCCATTCTTAATGCTGCATTA
M.brandtii    ATTAAGGTATG----ACCTAGTTGTTGTGAACGATGCATTCCATTCTTAATGCCGAATTA
M.lucifugus   ATTAAGGTATG----ACCTAGTTGTTGTGAACGATGCATTCCATTCTTAATGCCGAATTA
E.fuscus      ATTAAGGTATTAACCACCTAGTTGTTGTGAATGATGCATTCCATTCTTAATGCCGCATTA
E.helvum      GTCATCCCAGTAAACACC-AGTATTGGGGTAC-AT-------------------------
P.vampyrus    GTCATCCCAGTAAACACC-AGTATGGAGGTAC-AT-------------------------
P.alecto      GTCATCCCAGTAAACACC-AGTATGGGGGTAC-AT-------------------------
P.parnellii   GGCGG--------------CGTGGGGGGGTCC-AT-------------------------


Consensus     GGGCCAATGAAC
M.davidii     AGGCCAATGGAC-> Filo-EVE continues
M.brandtii    GGGCCAATGAAC-> Filo-EVE continues
M.lucifugus   GGGCCAATGAAC-> Filo-EVE continues
E.fuscus      GGGCCAATAGAC-> Filo-EVE continues
E.helvum      ------------
P.vampyrus    ------------
P.alecto      ------------
P.parnellii   ------------


b) Predicted amino acid sequences of orthologous filovirus NP-related EVEs


MARV/Mt.Elgon-Mus   SIISNSVGQTRFSGLLIVKTVLEFILQKTDSGVTLHPLVRTSKVKNEVASFKQALSNLARHGE
SUDV/Gul-808892     TVISNSVAQARFSGLLIVKTVLDHILQKTDLGVRLHPLARTAKVKNEVSSFKAALGSLAKHGE
RESTV/Phi89-Penn    AVIANSVAQARFSGLLIVKTVLDHILQKTDQGVRLHPLARTAKVRNEVNAFKAALSSLAKHGE
BDBV/But-811250     AVIANSVAQARFSGLLIVKTVLDHILQKTEHGVRLHPLARTAKVKNEVSSFKAALASLAQHGE
EBOV/Yam-May        AVISNSVAQARFSGLLIVKTVLDHILQKTERGVRLHPLARTAKVKNEVNSFKAALSSLAKHGE
Consensus           TVMAGAVEQSRFSGLFMAKNLLTYIITNEGGAIKIHPLAQDRWTKEEMTPCESCVRNIPNHGI
M.davidii           TVMAGAVEQSRFSG?LMAKSVLTYIITNEGGAIKRHPPAQDRQTKEEMARFESCVRNIPNHGI
M.brandtii          TVMAGAVEQSRFSGLFMAKNLLTYIITNEGGAIKIHPLARDRWTKEEMTPCESCVRNIANHGI
M.lucifugus         TVMASAVEQSRFSGLFMAKNLLTYIITNEGGAIKIHPLAQDRWTKEEMTSCESCVRNIPNHGI


MARV/Mt.Elgon-Mus   YAPFARVLNLSGINNLEHGLYPQLSAIALGVATAHGSTLAGVNVGEQYQQLREAAHDAEVKLQ
SUDV/Gul-808892     YAPFARLLNLSGVNNLEHGLYPQLSAIALGVATAHGSTLAGVNVGEQYQQLREAATEAEKQLQ
RESTV/Phi89-Penn    YAPFARLLNLSGVNNLEHGLYPQLSAIALGVATAHGSTLAGVNVGEQYQQLREAATEAEKQLQ
BDBV/But-811250     YAPFARLLNLSGVNNLEHGLFPQLSAIALGVATAHGSTLAGVNVGEQYQQLREAATEAEKQLQ
EBOV/Yam-May        YAPFARLLNLSGVNNLEHGLFPQLSAIALGVATAHGSTLAGVNVGEQYQQLREAATEAEKQLQ
Consensus           YAPFARVLGLPGVAQIRHGPFPHLPAIALGVSAVHQSTPAGVNTDTRYQAFKETAHLA--QLE
M.davidii           YAPFARVLGLPGVARIEHGPFPHLPAIALGVSAVHQSTPAGVNTDTQYQALKEAAHQV--ELE
M.brandtii          YAPFARVFGLPGVAQIRHRPFPRLPAIALGVSAVHQSTPVGVNTDTRYQAFKETAHLA--QLE
M.lucifugus         YAPFARVLGLPGVDQIRHGPFPHLRAIALGVSAVHQ?TPA??STDTRYQAFKETAHLA—-QLE


c) 3' flanking site


                                                 -> End of Filovirus NP
Consensus     GAGATCAACCCAGTGTAGACCAGTAGGGGGGGTCCATAAGTGAGAAAGAGAAGGAAAGAA
M.davidii     GAGATCAACCTAGTGTAGACTTGTAGGGGGG-TCCATAAGAGAGAAAGAGAAGGAAAGAA
M.brandtii    GAGATCAACCCAATGTACACCAGTAGGAGGG-GCCATAAAAGAGAAAGAGAAGGAAAGAA
M.lucifugus   GAGATCAACCCAGTGTACACCAGTAGGGGGG-GCCATAGGAGAGAAAGAGAAGGAAAGAA
E.fuscus      GAGATCAACCCAGTGTATACCAGTAGGGGGGGTCCATAAGTGAGAAAGAGAAGGAAAGAA
E.helvum      -------------------------------------AAGTGAGGCAGAGAAGGGAAGGA
P.vampyrus    -------------------------------------AAGTGAGGCAGAGAAGGAAAGGA
P.alecto      -------------------------------------AAGTGAGGCGGAGAAGGAAAGGA
P.parnellii   -------------------------------------AAGTGGGAAAGAGAAGGAAAGAA

[             421       431       441       451       461       471       ]
[             |         |         |         |         |         |         ]
Consensus     AGCTATAAAGGGGTGCTATCAAGCAAATGGACAACAGGATCTTAATCCCTCCAGGGAACT
M.davidii     AGCCACTCAGGGGTGCTCTCATGCAAATGGACAACAGGATCTTAATCCCTCCGGGGAACT
M.brandtii    AGCCATCCAGGGGTTCTCTCATGCAAATGGACAACAGGATCTCAATCCCTCCAGGGAACT
M.lucifugus   AACCATCCAGGGGTTCTCTCATGCAAATGGACAACAGGATCTCAATCCCTCCAGGGAACT
E.fuscus      AGCCACCCAGGGGTGCTCTCATGCAAATGGACAACAGGATCTCAATCCCTCCAGGGAACT
E.helvum      AGCTATAAAGGGGTGCTATCAAGCAAGTGGACAACTGGA--TTAACTGC-CCAGGGAACT
P.vampyrus    AGCTATAAAGGGATGCTATCAAGCAAATGGACAACTGGA--TTAATCCC-CCGAGGAACT
P.alecto      AGCTATAAAGGGGTGCTATCAAGCAAATGGACAACTGGA--TTAATCCC-CCGGGGAACT
P.parnellii   AGCTATAAAGGGGTGCTCTCAAGCAAGTGGACAGCAGGAGCTCAGTCCCTCCAGGGAACT

[             481       491       501       511       521       531       ]
[             |         |         |         |         |         |         ]
Consensus     CTGGGAAACAATACAGAACACCCCAGAGTATTCCTACCCAAGGGCGGGGAGCTGGGGGTA
M.davidii     CTGGGAAATAGTGCAGGATACCCCATGGTGATCCTACCCAAGGTTGGGGAGCTGGGAG-A
M.brandtii    CTGGGAAACAGTGCAGGATACCCCATGGTGATCCTACCCAAGGTTGGGGAGCTGGGAG-A
M.lucifugus   CTGGGAAACAGTGCAGGATACCCCATGGTGATCCTACCCAAGGTTGGGGAGCTGGGAG-A
E.fuscus      CTGGGAAACAGTGCAGGATACCCCATGGTGATCCTACCCAAGATTGGGGCGCTGGGGG-A
E.helvum      CTGGGAAACAATACAGAACACCCCAGAGTATTTCTATCTGAGGTTGGGGAGCTGGGGGTA
P.vampyrus    CTGGGAAACAATACAGAGAACCCCAGAGTATTGCTATCTGAGGGCGGGGAGCTGGGGGTA
P.alecto      CTGGGAAACAATACAGAGCACCCCAGAGTATTGCTATCTGAGGGCGGGGAGCTGGGGGTA
P.parnellii   CTGGGAAATAGTACCGAACACCC-GGGGTAT-CCTACCCAAGGGCGGGGAACTGGGGC-A

[             541       551       561       571       581       591       ]
[             |         |         |         |         |         |         ]
Consensus     GTTATCTACCATCTACCATCAGTCCTTGTCTAAGGGCTGCTGGGGGCAGGTGGTTAATTC
M.davidii     TTTGTCCGCCATCTACTATCAGTCCTTGTGTAA-GGCTGCTGGGG-AGGGTGTTTACTTC
M.brandtii    TTTGTCCACCATCTACCATCAGTCCTTGTGTAAGGGCTGCTGGGG-AGGGTGTTTACTTC
M.lucifugus   TTTGTCCACCATCTACCATCAGTCCTTGTGTAAGGGTTGCTGGGG-AGGATGTTTACTTT
E.fuscus      TTTACCCCCCACCTACCATCAGTCCTTGTGTAAGGGCTGCTGGGG-AGGGTGTTTACTTC
E.helvum      GTTATCTACCATCTATCTTCAGTCCTTGTCTAAGGGCTGCCGGGGTCAGGTGGTTAATTC
P.vampyrus    GTTATCTACCATCTACCTTCAGTCCTTGTCTAAAGGCTGCTGGGGGCAGGTGGTTAATTC
P.alecto      GTTATCTACCATCTACCTTCAGTCCTTGTCTAAAGGCTGCTGGGGGCAGGTGGTTAATTC
P.parnellii   TTTATCTGCCACCT-TCATCACTCCTTGTCTAAGGGCTGCTGGGGGCGGGTGGTGAATTC

[             601       611       621       631       641    ]
[             |         |         |         |         |      ]
Consensus     CCCACGGGCAGGCAAAAGCAGCTTCAGTGTTCAGAGAAAGCTCTAGG
M.davidii     CCCATGGGCAGGTAAAGGGGGCTTCTGTGTTCAGAGGAAGCTCTAGG
M.brandtii    CTCATGGGCTGGTAAAGGGGGCTTCCATGTTCAGAGGAAGCTCTAGG
M.lucifugus   CTCATGGGCAGGTAAAGGGGGCTTCTGTGTTCAGAGGAAGCTCTAGG
E.fuscus      CCCATGGGCAGGTAAAGGGAGCTTCCGTGTTCAGAGAAAGCTCTAGG
E.helvum      CCCACGGGCAGACAAAAGCAGCTTCAGTGTTCAGAGAAAGCTGTAGG
P.vampyrus    CCCACGGGCAGGCAAAAGCAGCTTCAGTGTTCAGAGAAAGCTCTAGG
P.alecto      CCCACGGGCAGGCAAAAGCAGCTTCAGTGTTCAGAGAAAGCTCTAGG
P.parnellii   CCCACGGGCAGGGAAAG-GGGGCTCAGTGTTCAGAGAACGCTCTGGG 
